# Controlling Crystal Orientation in Films of Conjugated Polymers by Tuning the Surface Energy

**DOI:** 10.1021/acs.macromol.4c01819

**Published:** 2024-10-29

**Authors:** Oleksandr Dolynchuk, Robert T. Kahl, Florian Meichsner, Alexander J. Much, Andrii Pechevystyi, Anna Averkova, Andreas Erhardt, Mukundan Thelakkat, Thomas Thurn-Albrecht

**Affiliations:** 1 Experimental Polymer Physics, Institute of Physics, Martin Luther University Halle-Wittenberg, Von-Danckelmann-Platz 3, Halle D-06120, Germany; 2 Applied Functional Polymers, University of Bayreuth, Universitätsstraße 30, Bayreuth D-95447, Germany; 3 Bavarian Polymer Institute, University of Bayreuth, Universitätsstraße 30, Bayreuth D-95447, Germany

## Abstract

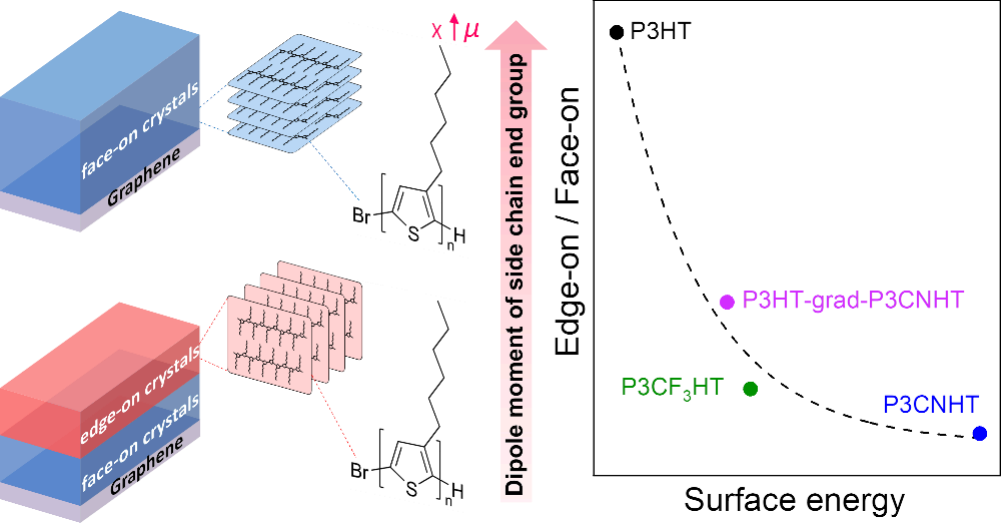

It has been a long-term goal to understand the molecular orientation in films of conjugated polymers, which is crucial to their efficient exploitation. Here, we show that the surface energies determine the crystal orientation in films of model conjugated polymers, substituted polythiophenes crystallized on substrates. We systematically increase the surface energy of edge-on crystals formed at the vacuum interface by attaching polar groups to the ends of the polymer side chains. This suppresses crystallization at the vacuum interface, resulting in a uniform face-on crystal orientation induced by the graphene substrate in polythiophene films as thick as 200 nm, which is relevant for devices. Surprisingly, face-on crystal orientation is attained in the modified polythiophenes crystallized even on amorphous surfaces. Furthermore, for the samples with still competing interfacial interactions, the crystal orientation can be switched in the same sample, depending on the crystallization conditions. Thus, we report a fundamental understanding and control of the equilibrium crystal orientation in films of conjugated polymers.

## Introduction

Conjugated polymers (CPs) are attractive materials for various electronic applications, which include organic field-effect transistors, organic light-emitting diodes, photovoltaics, organic electrochemical transistors, and so forth.^1−5^ The vast majority of CPs share the same chemical architecture consisting of the π-conjugated aromatic core and alkyl-type side chains attached to the core to enhance polymer solubility. While the charge transport along the CP backbones is limited by the chain length and thus the polymer molecular weight, the charge transport along the π–π stacking direction in the ordered crystalline or liquid crystalline CPs extends far beyond the chain length, making this molecular direction highly desirable for applications.^5^ The electronic properties in films of CPs depend primarily on several key parameters, which include HOMO and LUMO energy levels, bandgap, and morphological features such as molecular packing in the crystal, crystallinity, and crystal orientation.^5−7^ The latter has been shown to significantly impact the charge carrier mobility.^8−11^ Thus, understanding what parameters govern the alignment and having a tool to control the crystal orientation would be a significant step forward in making organic electronic devices more efficient and elucidating the exact role of the aforementioned key parameters in the electronic properties of CPs.

**Figure 1:**
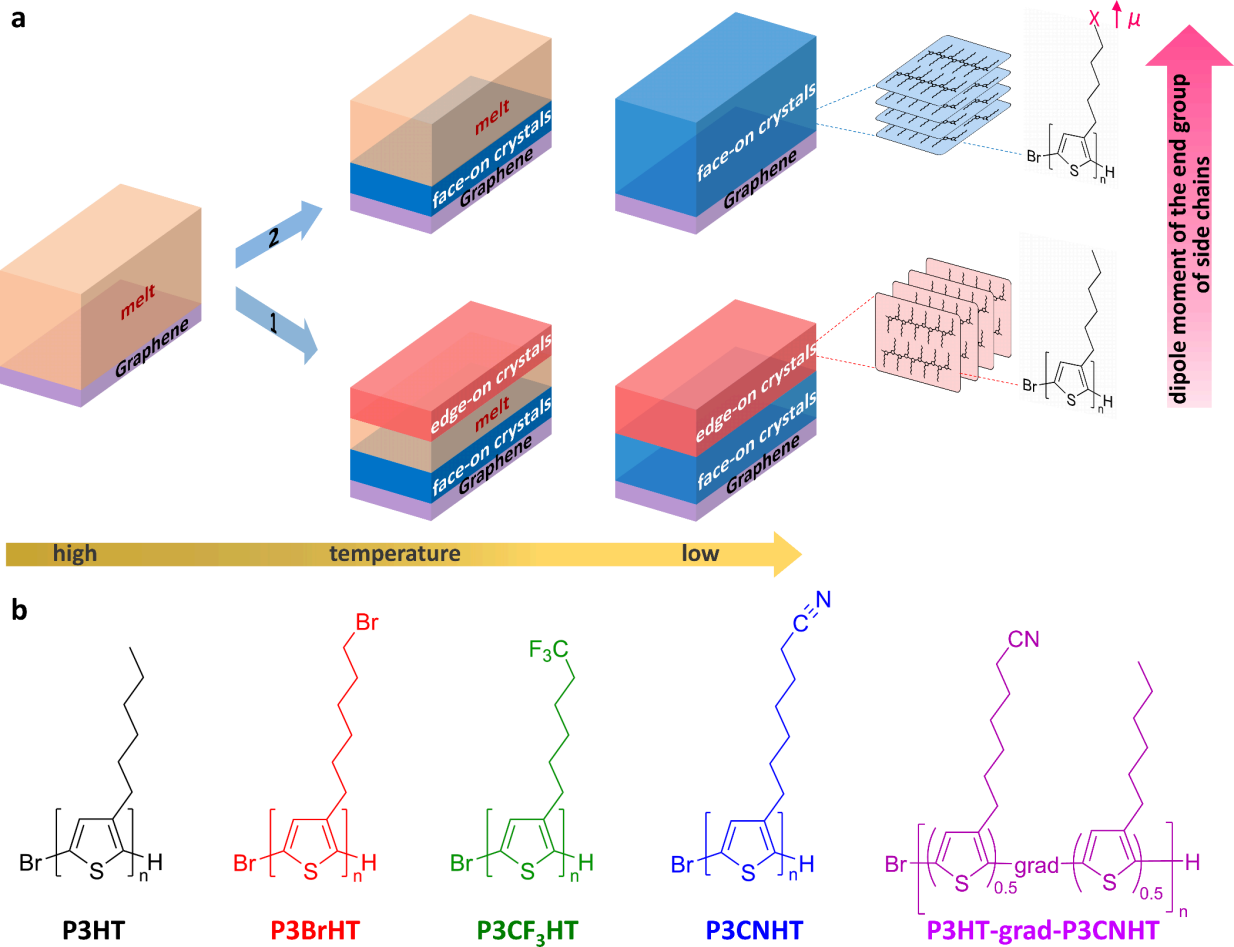
Schematic illustration of the crystallization of a series of P3ATs films on graphene and their chemical structures. (a) The molten P3AT film is indicated in orange. Upon cooling the P3HT film (pathway 1), the edge-on oriented crystals (red) form at the interface to the vacuum, while the face-on oriented crystals (blue) form at the interface to graphene. After crystallization is complete, the P3HT film has a double-layered out-of-plane morphology consisting of edge-on- and face-on oriented crystals. When cooling films of P3ATs with the increased dipole moment of the end group of side chains (pathway 2), the crystallization at the vacuum interface is suppressed, which promotes the growth of face-on crystals induced by the interface to graphene into the entire film. (b) The chemical structures and names of P3ATs used in this work to study the influence of increasing dipole moment of the end group of side chains on crystal orientation.

Poly(3-hexylthiophene) (P3HT) is a well-studied p-type CP with high crystallinity, chemical, and thermal stability.^3−5^ Although P3HT is not currently the most promising CP for applications, it has a sufficiently simple molecular structure and is, therefore, the most appropriate CP to address the fundamental question of how to gain control over the crystal orientation in CPs. The so-called face-on molecular orientation in films of P3HT and other CPs with the vertically π–π stacked chains (Figure 1a) is particularly attractive for applications requiring vertical charge transport, such as organic photovoltaics.^5,9,10^ Methods such as mechanical stretching and rubbing,^12−15^ the use of a low-boiling solvent,^16^ and specific substrates have recently been exploited to induce the face-on orientation in P3HT and other π-conjugated oligomers and polymers.^17−27^ These approaches have only been partially successful, as complete face-on orientation in P3HT films with thicknesses of 100 nm or more, relevant for organic electronic devices, has not yet been achieved. Furthermore, with the exception of the crystallization process from the melt on substrates, the aforementioned methods result in nonequilibrium crystal structures with relatively low thermal stability. These crystals are likely to reorganize upon heating, causing an irreversible change in the molecular orientation. Although the CP films are typically supported on substrates, the influence of the substrate on the molecular orientation can be obscured by other factors, including the rate of solvent evaporation, spinning speed, mechanical strain, and so forth. In contrast, oriented crystallization from the melt on substrates eliminates these disadvantages and is readily used to orient inorganic solids in- and out-of-plane.^28^ In general, anisotropic crystal orientation in semicrystalline polymer films crystallized on substrates is a result of interface-induced crystallization.^29−33^ The latter can be either heterogeneous nucleation,^33^ prefreezing at the substrate interface,^29−32,34^ or surface freezing at the interface to vacuum or air.^35−38^ While heterogeneous nucleation is an activated process that results in the formation of crystal nuclei at the interface to a solid material below melting temperature *T*_m_,^33,39,40^ prefreezing and surface freezing are equilibrium phenomena occurring above *T*_m_.^29−38^ These crystallization phenomena share a common feature, their occurrence and the properties of the resulting ordered phase, including crystal orientation, are strongly dependent on the surface and interfacial energies γ, according to the theory.^39,40^ As reported in the previous works, the crystal orientation in CPs is indeed influenced by the interfaces, not only those to the substrates but also the “free” interface to the vacuum.^41,42^ It has been shown that the upper interface to the vacuum in P3HT films, regardless of the substrate, induces the formation of edge-on oriented crystals with the side chains normal to the film surface (Figure 1a). This discovery allowed explaining the limited influence of graphene on the face-on orientation of P3HT crystals as a result of the competition between the interfaces to graphene and vacuum and suggested that the influence of any substrate used to orient the P3HT crystals may be fundamentally limited.^42^ Therefore, to control the crystal orientation in P3HT films, it is necessary to influence the surface energies of the polymer at the interface to vacuum to suppress the edge-on crystallization at this interface and achieve a uniform orientation induced by the substrate. Polymer surface energies can be tuned by changing the dipole moment μ of the repeating units through chemical modification of CPs.^43−45^ Considering the important role of the conjugated aromatic cores for the electronic properties, we recently proposed to chemically modify the dipole moment of the end group of the side chains of P3HT to influence the surface energies of edge-on crystals γ_edge_ and, in turn, the crystal orientation in P3HT films.^42^ This idea was tested in poly-(3-(6-bromohexyl))thiophene (P3BrHT) films on graphene and found to be valid, although the observed effect was limited to thin films with a thickness of up to 26 nm, raising the question of whether stronger effects can be achieved in general. In addition, the correlation between surface energies and crystal orientation, which is central to our strategy, was not quantified and remained speculative.

Here, we present an extension and thorough verification of this strategy and report the synthesis of a series of poly-3-alkylthiophenes (P3ATs) with the polar end group of the side chains bearing high dipole moments μ (Figure 1b). To allow for a larger set of samples, we also utilized the copolymerization of 3-alkylthiophenes with end groups of different μ, which has recently been shown to be a successful approach to tune the surface energies in P3ATs.^43^ Our results confirm that the surface energy of edge-on crystals γ_edge_ increases with increasing dipole moment μ. The crystal orientation formed in these samples after crystallization from the melt on graphene and the large- and small-scale morphologies are carefully analyzed using grazing-incidence wide-angle X-ray scattering (GIWAXS), atomic force microscopy (AFM), and optical microscopy (OM) and show direct correlation with γ_edge_. Furthermore, we investigate the influence of the introduced chemical modification on the crystal orientation formed in the modified P3ATs on a silicon substrate, which has no impact on crystallization of P3HT. Finally, we demonstrate that when competition between different interfaces is still present in P3ATs films, crystallization kinetics plays an important role in crystal orientation and can be used to control it.

## Experimental Section

### Materials and Synthesis

P3HT was purchased from BASF SE. P3HT was purified by a Soxhlet extraction using methanol, hexane, and chloroform. The chloroform fraction was concentrated and precipitated in methanol to obtain the polymer powder used in our experiments. The detailed description of synthesis of P3BrHT, poly-(3-(6-trifluorohexyl))thiophene (P3CF_3_HT), poly-(3-(6-cyanohexyl))thiophene (P3CNHT), and poly-(3-hexylthiophene)-gradient-poly-(3-(6-cyanohexyl))thiophene (P3HT-grad- P3CNHT) can be found in the Supporting Information. All reactions were carried out under the exclusion of moisture and air, under an argon inert gas atmosphere. Dry solvents, Grignard reagent, and catalyst were purchased from Acros Organics, and KCN was purchased from Sigma-Aldrich. Trimethoxypropylsilane, trimethoxy(3-bromopropyl)silane, and trimethoxy(3,3,3-trifluoropropyl)silane were purchased from TCI, and trimethoxy(3-cyanopropyl)silane was purchased from abcr GmbH.

Single-layer graphene on the ultraflat SiO_2_/Si 5 × 5 mm^2^ diced substrates produced by Ted Pella, Inc. was purchased from PLANO GmbH and used for films of P3HT and P3BrHT. Single-layer graphene on the ultraflat SiO_2_/Si 10 × 10 mm^2^ diced substrates was purchased from Graphenea, Inc. and used for films of P3CF_3_HT, P3HT-grad-P3CNHT, and P3CNHT.

The silicon substrates were cut from a silicon wafer with a naturally oxidized SiO_2_ layer of 2–3 nm. The resulting substrates were approximately 10 × 10 mm^2^. The substrates were cleaned in sulfuric acid for 30 min. After being rinsed with distilled water, the substrates were heated to 160 °C in a vacuum oven and kept at this temperature for 30 min. Directly before spin coating, the substrates were cleaned with a CO_2_-snowjet.

### NMR Spectroscopy

All proton NMR spectra were recorded in CDCl_3_ on a Bruker Avance 250 spectrometer with a frequency of 300 MHz. All spectra were calibrated to the CDCl_3_ signal at 7.26 ppm.

### Size Exclusion Chromatography (SEC)

SEC was performed utilizing a Waters 515 HPLC pump with stabilized THF as an eluent at a flow rate of 0.5 mL·min^–1^. A volume of 100 μL of polymer solution (1–2 mg·mL^–1^) was injected with a 707 Waters autosampler into a column setup comprising a guard column (ResiPore Guard, 5 × 0.75 cm, particle size 3 μm) and two separation columns (ResiPore, 30 × 0.75 cm, particle size 3 μm). Polymer size distributions were monitored with a Waters 998 photodiode array detector at 254 nm and a Waters 414 refractive index detector. Narrow distributed polystyrene standards were used for calibration and 1,2-dichlorobenzene as an internal reference.

### Matrix-Assisted Laser Desorption/Ionization Time-of-Flight Mass Spectrometry (MALDI-ToF MS)

MALDI-ToF MS measurements were performed by using a Bruker AutoFlex Max mass spectrometer equipped with a Smartbeam II laser. The analyte was embedded in the matrix material *trans*-2-[3-(4-*tert*-butylphenyl)-2-methyl-2-propenylidene]malononitrile (DCTB) at a 10:1 matrix:analyte mass ratio.

### Infrared (IR) Spectroscopy

IR transmission spectra were recorded with a PerkinElmer Spectrum 100 FTIR-spectrometer in ATR mode.

### Thin Film Preparation

Thin films of semiconducting polymers were prepared by spin-coating P3HT, P3BrHT, P3HT-grad-P3CNHT, and P3CNHT solutions in chloroform and P3CF_3_HT solutions in THF onto graphene and silicon substrates at 2000 rpm for 60 s at room temperature. The concentration of polymers in the solutions varied from 0.25 to 1.5 wt% to obtain thin films with different thicknesses. The film thickness was determined either by X-ray reflectivity or by scratching the films with a surgical knife and measuring the depth of the resulting scratch with AFM. The spin-coated films of P3HT on silicon and P3HT-grad-P3CNHT, P3CF_3_HT, and P3CNHT on silicon and graphene were crystallized by cooling from the melt in a vacuum on the Linkam hot stage HFS350 directly in the GIWAXS setup. The complete melting of the polymer films was verified by GIWAXS measurements at high temperatures above the corresponding melting temperatures of the polymers. Unless otherwise stated, the films were cooled down at 1 °C·min^–1^ to 120 °C, below the corresponding crystallization temperatures, and then cooled down at 10 °C·min^–1^ to 20 °C. The spin-coated films of P3HT and P3BrHT on graphene were crystallized from the melt in a vacuum oven after heating to 285 °C for P3HT and 157 °C for P3BrHT for 5 min and then slowly cooling down (≈1 °C min^–1^) to room temperature.

### Contact Angle Measurements

Sessile drop contact angle measurements were performed on the P3HT, P3BrHT, P3HT-grad-P3CNHT, P3CF_3_HT, and P3CNHT films on silicon with a thickness of 20–30 nm at room temperature with bidistilled water. The water droplets had a volume of approximately 1.5 μL. The droplet contact angles were monitored using the OCA 20 optical contact angle measuring system from DataPhysics Instruments GmbH. The obtained values of the droplet contact angles were converted into the surface energies using the method of Neumann.^46^ Prior to the measurements, all polymer films were crystallized from the melt and all showed an edge-on crystal orientation in their top layer (Figure S6). The P3HT, P3BrHT, and P3CNHT films on silicon used were crystallized in a vacuum oven, while the P3HT-grad-P3CNHT and P3CF_3_HT films were crystallized on a Linkam hot stage THMS600 directly in the GIWAXS setup.

### WAXS and GIWAXS

The WAXS and GIWAXS experiments were performed using a SAXSLAB laboratory setup (Retro-F) (Copenhagen, Denmark) equipped with an AXO microfocus X-ray source (Dresden, Germany) and an AXO multilayer X-ray optics (ASTIX) as a monochromator for Cu Kα radiation (λ = 0.15418 nm). A DECTRIS PILATUS3 R 300K detector (Daettwil, Switzerland) was used to record the 2D GIWAXS patterns. The powder and thin film measurements were performed in transmission and reflection geometry, respectively, in a vacuum at room temperature, and the sample-to-detector distance was around 92 mm. The GIWAXS detector images were converted into the reciprocal space maps with two components, *q*_z_ and *q*_p_, being perpendicular and parallel to the film surface, respectively. Due to the special geometry of the measurements, a certain area of the reciprocal space along the *q*_z_ axis was not accessible and appeared as a blank arc. Two additional blank vertical strips arose at the positions where two of three adjacent parts of the detector meet and were inactive regions of the detector.

### OM

The OM images were taken with an Olympus BX51 microscope. The magnifications were either 50× or 200×, depending on the objective chosen. All images were taken in a reflection geometry.

### AFM

AFM images were recorded in peak force tapping mode using a Bruker MultiMode 8 AFM with a Nanoscope V controller equipped with a ScanAsyst–Fluid+ cantilever (*f*_0_ = 150 kHz, *k* = 0.7 N·m^–1^) purchased from Bruker. The cantilever was operated at an excitation frequency of 2 kHz. For the discussion of the surface morphology, height and adhesion images were used. Adhesion images are a measure of the adhesive forces between the tip and the sample surface during tapping. The open-source software Gwyddion was used to edit and analyze the AFM images.^47^

## Results and Discussion

**Figure 2:**
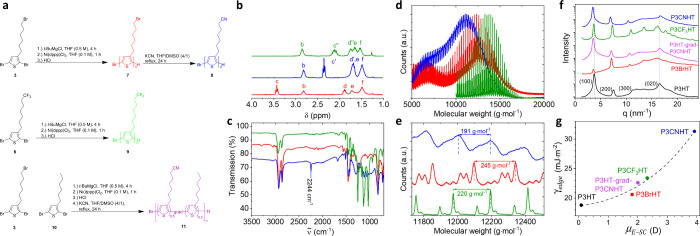
Synthesis and bulk properties of P3ATs with different end groups on the side chains. (a), Synthesis of P3BrHT, P3CNHT, P3CF_3_HT, and P3HT-grad-P3CNHT investigated in this work. (b) Aliphatic region of the NMR spectra of P3BrHT, P3CNHT, and P3CF_3_HT. (c) IR spectra of P3BrHT, P3CNHT, and P3CF_3_HT. A pronounced band at 2244 cm^–1^ in the P3CNHT spectrum, which is typical for the C≡N valence vibration, can be observed, indicating a successful polymer analogous substitution. (d) MALDI-ToF spectra of P3BrHT, P3CNHT, and P3CF_3_HT. (e) MALDI-ToF spectra of P3BrHT, P3CNHT, and P3CF_3_HT show a peak distance of 245 g·mol^–1^ (rep. unit of P3BrHT), 191 g·mol^–1^ (rep. unit of P3CNHT), and 220 g·mol^–1^ (rep. unit of P3CF_3_HT). (f) WAXS patterns of powder samples of P3HT, P3BrHT, P3CNHT, P3CF_3_HT, and P3HT-grad-P3CNHT. (g) Dependence of the surface energy of edge-on crystals γ_edge_ of P3ATs under study on the dipole moment of small molecules μ_E–SC_ equivalent to the end group of P3ATs side chains: ethane for P3HT, methyl bromide for P3BrHT, 1,1,1-trifluoroethane for P3CF_3_HT, and acetonitrile for P3CNHT.^49^ The value of μ_E–SC_ in the case of P3HT-grad-P3CNHT was estimated as the average of μ_E–SC_ for ethane and acetonitrile.

Figure 2a shows the synthesis of P3ATs with different end groups on their side chains. We started from the functional monomer 2,5-dibromo-3-(6-bromohexyl)thiophene and prepared the ω-substituted P3BrHT, which was recently presented by us.^48^ P3BrHT served as the precursor polymer for the novel poly-(3-(6-cyanohexyl))thiophene (P3CNHT); therefore, P3BrHT was converted to P3CNHT in a polymer analogous substitution (S_N_2) reaction with KCN (Figure 2a). The novel second polymer poly-(3-(6,6,6-trifluorohexyl))thiophene (P3CF_3_HT) was synthesized from 2,5-dibromo-3-(6,6,6-trifluorohexyl)thiophene. Additionally, we synthesized the gradient copolymer P3HT-grad-P3CNHT with a 50/50 composition. Since the CN group has the largest and the CH_3_ group the smallest dipole moments among those used in this work, the copolymerization of 3HT and 3CNHT monomers allowed obtaining a P3AT with the mean dipole between those of P3HT and P3CNHT. We first prepared the recently reported precursor polymer P3HT-grad-P3BrHT and converted it to P3HT-grad-P3CNHT in a polymer analogous substitution reaction equivalent to the synthesis of the homopolymer P3CNHT (Figure 2a).^48^ All mentioned P3ATs were synthesized via Kumada catalyst transfer polymerization (KCTP), which is a versatile tool for the controlled synthesis of P3ATs, yielding polymers with controlled molecular weight, low dispersity, high regioregularity, and defined end groups. The ^1^H NMR spectra of P3CNHT in Figure 2b show a strong and complete high field shift of the protons of the CH_2_ group in direct vicinity to the functional group of the side chain and, thus, prove successful chemical reactions. The full NMR spectra of all synthesized and investigated polymers are shown in the Supporting Information (Figures S1 and S2). Moreover, the IR spectrum of P3CNHT (Figure 2c) evidenced an additional vibrational band at υ̃ = 2244 cm^–1^, which is present in IR spectra of neither the precursor polymer P3BrHT nor P3CF_3_HT and can be assigned to the valence oscillation of the CN group proving the successful polymer analogous substitution. The molecular weight and polydispersity were determined by SEC and MALDI-ToF mass spectrometry (Figure S3). MALDI-ToF was used to determine the absolute molecular weight and the degree of polymerization for the investigated polymers (Figure 2d,e, Table 1). Here, it is to mention that for P3HT, only the low molecular weight fraction of the polymer could be detected in MALDI-ToF (Figure S3), which might be due to the relatively high dispersity of the P3HT sample leading to mass discrimination effects. The molecular weight for MALDI-ToF and the number of repeating units therefore is calculated from a MALDI-SEC-correlation curve published elsewhere.^48^ MALDI-ToF measurements revealed a comparable degree of polymerization around 50–60 repeating units for the investigated polymers (Table 1). Note that for P3HT-grad-P3CNHT, no degree of polymerization could be determined because the ionized polymer did not fly and, therefore, could not be detected in our MALDI-ToF experiments. All novel polymers P3CF_3_HT, P3CNHT, and P3HT-grad-P3CNHT exhibit chemical stability up to 350 °C (Figure S4) and similar values of *T*_m_ ranging from 190 to 212 °C (Figure S4, Table 1).

**Table 1:** Molecular Weight and Bulk Properties of P3ATs with Different End Groups on the Side Chains^a^

**polymers**	***M*_n_**^b^ (kDa)	***Đ*^b^** (-)	**DP^c^** (-)	***T*_m_^d^** (°C)	**Δ***H*_m_^d^ (J·g^–1^)	**χ**_c_^e^ (%)	***d*_100_^e^** (nm)	***d*_020_^e^** (nm)	**μ**_E__–__SC_ (D)	**γ**_edge_^f^ (mJ·m^–2^)
P3HT	15.6	1.6	60	231	24.6	81	1.68	0.38	∼0	18.8
P3BrHT	10.8	1.1	50	136	7.1	43	1.77	0.388	1.81	20.6
P3HT-grad-P3CNHT	23.6	1.28		196	13.6	62	1.78	0.38	∼2	22.6
P3CF_3_HT	22	1.26	56	212	18	67	1.73	0.389	2.32	23.4
P3CNHT	10.3	1.56	58	190	10.2	52	1.82	0.376	3.92	31.3

a Molecular weight *M_n_,* dispersity *Đ*, degree of polymerization DP, melting temperature *T*_m_, melting enthalpy Δ*H*_m_, crystallinity χ_c_, interlayer distances *d*_100_ and *d*_020_, dipole moment of small molecules μ_E–SC_ equivalent to the end group of P3ATs side chains,^49^ and surface energy of edge-on crystals γ_edge_.

b Determined by SEC.

c Determined by MALDI-ToF.

d Determined by DSC.

e Determined by WAXS. The values of χ_c_ were obtained from the WAXS measurements according to the procedure described elsewhere.^48^

f Determined by contact angle measurements.

The powder wide-angle X-ray scattering (WAXS) curves for all P3ATs (Figure 2f) are quite similar, featuring equidistant (h00) crystal reflections from the periodicity of backbones in the direction of side chains and a (020) reflection from the π–π stacked thiophene rings. Note that the intensity of the (h00) peaks differs between the P3ATs due to the different electron density distribution along the side chain direction, with the largest differences in P3BrHT.^48^ The interlayer distances *d*_100_ and *d*_020_, as determined from the WAXS curves in Figure 2f, together with the unit cell parameters derived from the GIWAXS patterns of thin oriented films (Table S1) reveal very similar chain packing, with the most notable differences for *d*_100_ in the direction of the side chains (Table 1). As shown in Figure S5, the polymer segments in P3HT-grad-P3CNHT cocrystallize and build a common crystal lattice, which is a prerequisite for further investigation. Thus, the crystal orientations formed in films of these different P3ATs can be readily compared and analyzed. In order to verify our central hypothesis of the correlation between the dipole moment of the end group on side chains and the surface energy of edge-on crystals γ_edge_, we determined γ_edge_ for all P3ATs under study by static contact angle measurements with water (Figure S6). Special care was taken to ensure that the top layer of P3ATs was edge-on oriented (Figure S6). Note that the edge-on orientation in P3CNHT film on silicon crystallized in a vacuum oven could not be reproduced during the controlled crystallization of this sample in the GIWAXS setup (see methods). The obtained results in Figure 2g clearly indicate that γ_edge_ increases monotonically from 18.8 to 31.3 mJ·m^–2^ with increasing dipole moment of small molecules μ_E–SC_ equivalent to the end group of P3ATs side chains (Table 1). Additional studies of the surface energies of the silane monolayers modified similarly to the side chains of P3ATs prove that the attachment of the CN group indeed confers the highest surface energy (Figures S7 and S8).

**Figure 3:**
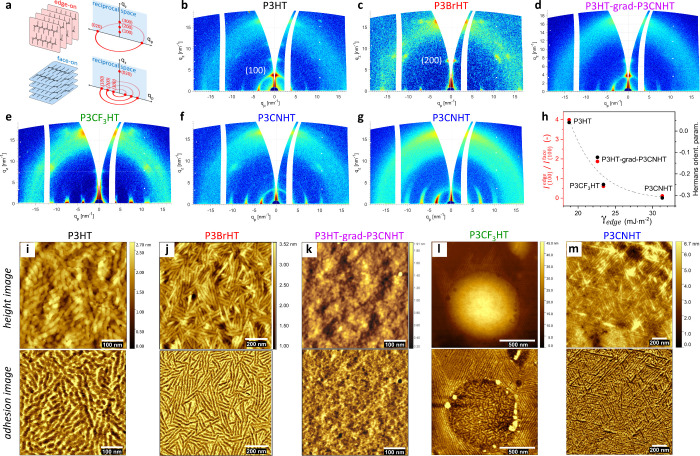
Influence of surface energy on the crystal orientation and surface morphology in P3AT films on graphene. (a) Sketches of the reciprocal space maps of edge-on and face-on crystals given that their orientation in the film plane is isotropic. (b–g) GIWAXS patterns of (b) 46 nm thick P3HT, (c) 46 nm thick P3BrHT, (d) 60 nm thick P3HT-grad-P3CNHT, (e) 55 nm thick P3CF_3_HT, (f) 44 nm thick, and (g) 193 nm thick P3CNHT on graphene measured at incident angles (b,d–g) 0.18° and (c) 0.2°, above the corresponding critical angles of the polymers, which are 0.174° for P3BrHT and 0.163–165° for other P3ATs. (h) Ratio of intensities  (red, left axis) and the Hermans orientation parameter (black, right axis) of the (100) reflection scattered from edge-on and face-on crystals of P3ATs on graphene as a function of the surface energy γ_edge_. The ratio  and the Hermanns orientation parameter were calculated from the corresponding plots of the azimuthal intensity distribution of the (100) reflection (Figure S10). (i–m) AFM height (up) and adhesion (bottom) images of (i) 46 nm thick P3HT,^42^ (j) 35 nm thick P3BrHT,^42^ (k) 60 nm thick P3HT-grad-P3CNHT, (l) 25 nm thick P3CF_3_HT, and (m) 21 nm thick P3CNHT on graphene. The imaged area varied and ranged between 1.5 × 1.5 and 0.5 × 0.5 μm^2^ to better visualize the features. The white scale bars in each image enable the size of the features to be estimated.

The influence of the surface energy γ_edge_ on the crystal orientation was investigated in films of P3ATs with a mesoscopic thickness of about 50–60 nm crystallized from the melt on graphene. The GIWAXS patterns in Figure 3b-g show the azimuthal distribution of the most intense (100) crystal reflection in all P3ATs except for P3BrHT with the most intense (200) reflection. While P3HT, P3BrHT, and P3HT-grad-P3CNHT clearly show mixed edge-on and face-on crystal orientation, P3CF_3_HT is predominantly face-on, and P3CNHT is fully face-on oriented. Surface-sensitive GIWAXS was used to investigate the distribution of the crystal orientation throughout the film thickness in P3HT-grad-P3CNHT on graphene (Figure S9) and confirmed that face-on crystals dominate at the interface to graphene, whereas edge-on crystals dominate at the interface to vacuum, similar to P3HT and P3BrHT studied earlier and in line with the model in Figure 1a.^42^ Note that only P3CNHT shows the absence of edge-on oriented crystals. The isotropic scattering signal of the (100) reflection in Figure 3f is split and, therefore, comes from the crystals located at the edges of the substrate. Although the amount of edge-on crystals in P3CF_3_HT is small, as indicated by the low intensity value of the (100) reflection scattered from edge-on crystals , it is not negligible and allows concluding that the edge-on orientation in P3CF_3_HT has been significantly suppressed but not completely. As introduced in our previous work,^42^ the ratio of the intensities scattered by the edge-on and face-on oriented crystals  is an appropriate parameter to evaluate the relative proportion of the respective crystal orientations. Indeed, it takes into account the different intensities of the (100) reflections and the different crystallinities of P3ATs (Figure 2f and Table 1), which would otherwise make a rational comparison between the samples impossible. Thus, the ratio  was obtained from the respective azimuthal intensity distributions of the (100) reflection (Figure S10) and is plotted versus the surface energy γ_edge_ in Figure 3h. Moreover, the Hermans orientation parameter, a widely used measure of crystal orientation, was also calculated and is plotted in Figure 3h. As the (100) reflection of P3BrHT has a very low intensity and cannot be detected and analyzed well in the corresponding GIWAXS pattern (Figure 3c), it was excluded from further analysis. Nevertheless, the monotonic decrease of both  and the Hermans orientation parameter with increasing γ_edge_ in Figure 3h provides compelling evidence that the amount of edge-on crystals is gradually decreasing, promoting the formation of more face-on oriented crystals in films of P3ATs with more polar side chains. This fully supports our strategy of suppressing edge-on crystals in P3ATs. To test whether the influence of graphene on the formation of face-on crystals can also be realized in thicker films, we spin-coated the P3CNHT film with a thickness of about 200 nm and crystallized it from the melt on graphene. The GIWAXS pattern of this sample after removing the sample edges (Figure 3g) shows a complete face-on orientation of polymer crystals, confirming that the formation of edge-on crystals was fully suppressed in P3CNHT. Thus, we demonstrate that the uniform out-of-plane crystal orientation induced by graphene can be achieved in films of P3ATs with thicknesses relevant for applications.

The surface morphology of the P3AT film crystallized on graphene was examined with AFM and is shown in Figure 3i–m. While the morphology of P3HT-grad-P3CNHT films shows small grain-like crystallites, the morphology of all other P3ATs is represented by lamellar crystals. As visible in Figure 3m, the lamellae of P3CNHT crystallize epitaxially on graphene. Thereby, the influence of the graphene substrate results not only in the out-of-plane face-on orientation of P3CNHT but also in the in-plane orientation of the crystalline lamellae. Figure 3l displays the dewetted morphology of the originally smooth 25 nm thick P3CF_3_HT film, which is also well visible for the 55 nm thick sample by optical microscopy (OM) (Figure S11). Note that after crystallization from the melt, the 25 nm thick P3CF_3_HT film dewetted and formed droplets with a mean height of about 85 nm. The droplet in Figure 3l is surrounded by the continuous polymer film consisting of long polymer lamellae crystallized epitaxially on the graphene substrate, similar to those in P3CNHT. This observation, on the one hand, confirms the direct influence of graphene on the crystallization of P3CF_3_HT and, on the other hand, suggests that the dewetting of P3CF_3_HT was authophobic, that is, the molten material dewetted on its own epitaxially aligned crystals.^29,31^ By contrast, the crystal morphology on the top of the droplet is represented by randomly oriented short lamellae. Remarkably, this surface morphology is very similar to that formed in P3HT and P3BrHT, where the top layer is edge-on oriented, as reported previously.^42^ So, we conclude that the edge-on crystals in P3CF_3_HT are formed at the interface to vacuum, while the face-on crystals form at the interface to graphene, quite congruent with the model in Figure 1a. Altogether, these observations confirm that the increased values of γ_edge_ in P3CF_3_HT and P3CNHT have a strong influence on the crystal orientation and morphology initiated by the underlying graphene substrate. It is natural to expect that this influence can also manifest in these P3ATs crystallized on other substrates. Since silicon is a commonly used substrate for films of CPs and has been shown to have no impact on the crystallization of P3HT films, it is interesting to test the influence of silicon on the crystallization of P3CF_3_HT and P3CNHT films.

**Figure 4:**
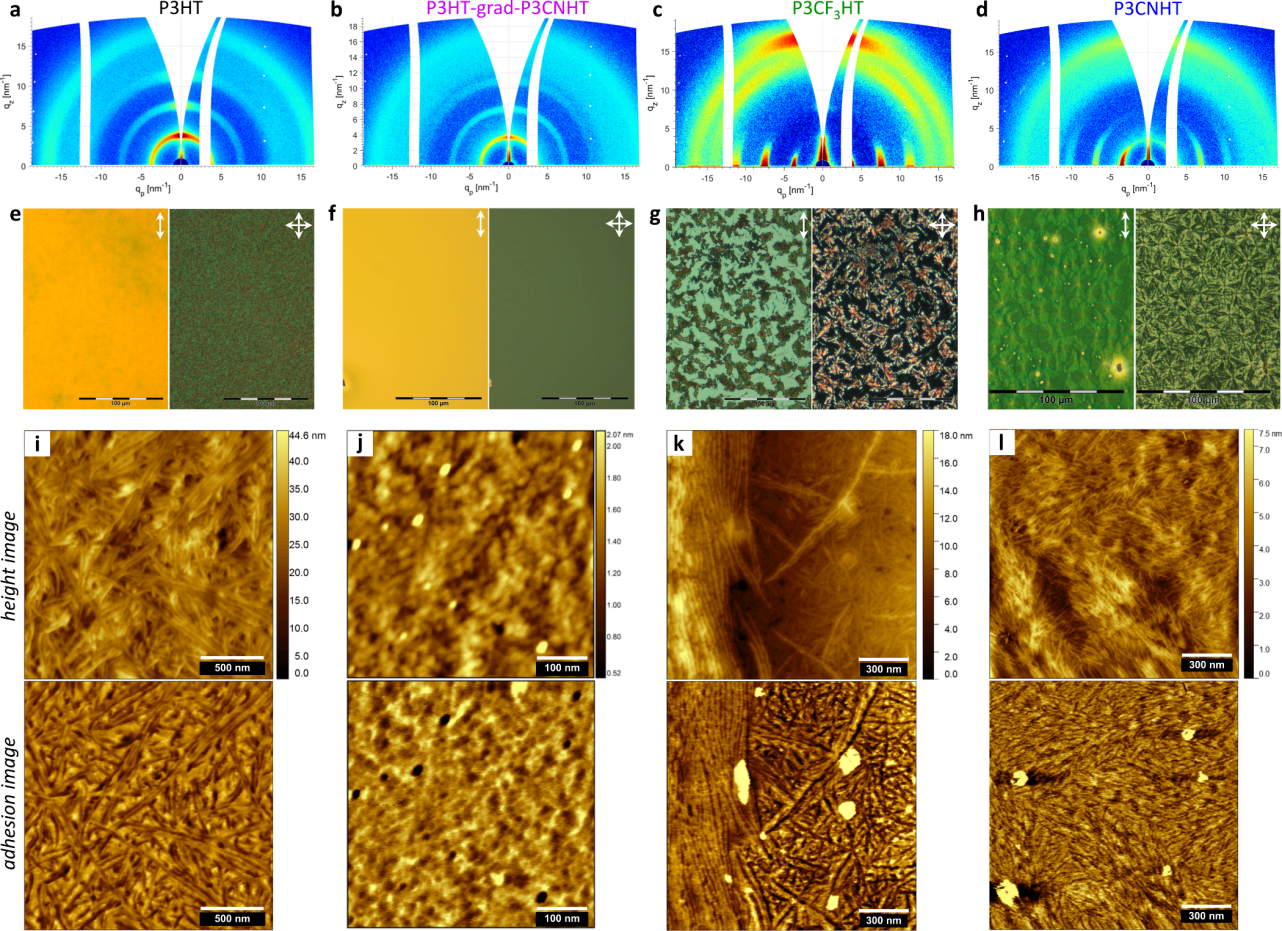
Crystal orientation and surface morphology of P3AT films crystallized on silicon. (a–d) GIWAXS patterns of (a) 120 nm thick P3HT, (b) 128 nm thick P3HT-grad-P3CNHT, (c) 85 nm thick P3CF_3_HT, and (d) 95 nm thick P3CNHT on silicon measured at an incident angle of 0.18°, above the corresponding critical angles of the polymers, which are 0.163–165°. (e–h) OM images of the corresponding films of (e) P3HT, (f) P3HT-grad-P3CNHT, (g) P3CF_3_HT, and (h) P3CNHT on silicon were taken with open (left half of the images) and crossed polarizers (right half of the images). The images with open and crossed polarizers were taken from the same area of the respective films and marked with the vertical and crossed white arrows, respectively, in the upper right image corners. (i–l) AFM height (up) and adhesion (bottom) images of of the corresponding films of (i) P3HT, (j) P3HT-grad-P3CNHT, (k) P3CF_3_HT, and (l) P3CNHT on silicon. The imaged area varied and ranged between 1.5 × 1.5 and 0.5 × 0.5 μm^2^ to better visualize the features. The white scale bars in each image enable the size of the features to be estimated.

Figure 4a,b presents the GIWAXS patterns of P3HT and P3HT-grad-P3CNHT films crystallized on silicon, which demonstrates the dominating edge-on crystal orientation and isotropic signal from unoriented crystals. Note that the intensity of the edge-on peak in P3HT-grad-P3CNHT is visibly lower than that of P3HT that suggests a decreased tendency for edge-on orientation. Astonishingly, the polymer crystals in both P3CF_3_HT and P3CNHT are strongly face-on oriented (Figure 4c,d). However, while the P3CNHT film is exclusively face-on oriented, the GIWAXS pattern of the P3CF_3_HT film shows a weak (100) peak from the edge-on crystals. While the OM images of P3HT and P3HT-grad-P3CNHT films are weakly birefringent (Figure 4e,f), the OM images of P3CF_3_HT and P3CNHT films in Figure 4g,h clearly show strong birefringence featuring Maltese cross patterns, which are typical for spherulitic macrostructures. The spruce-like arrangement of the P3CNHT lamellae in Figure 4l confirms the spherulitic crystal growth. Considering that the polymer crystals inside the spherulites are face-on oriented, it can be concluded that both P3CF_3_HT and P3CNHT crystallize on silicon via heterogeneous nucleation.^33,39^ As seen in Figure 4g, P3CF_3_HT has partially dewetted during crystallization and formed nonbirefringent islands on the silicon substrate. The AFM study performed on the border between the birefringent and nonbirefringent regions (Figure 4k) reveals that the nonbirefringent regions consist of polymer lamellae with no preferred in-plane orientation, similar to those observed on the top of the dewetted P3CF_3_HT droplets on graphene and those of P3HT and P3HT-grad-P3CNHT in Figure 4i,j. The additional study of the thickness dependence of the crystal orientation and morphology of P3CF_3_HT on silicon (Figures S12–S14) confirms that the thin polymer film in the nonbirefringent regions in Figure 4g adopts an edge-on crystal orientation. All in all, these experimental outcomes allow for the conclusion that the edge-on and face-on crystal orientations in P3CF_3_HT are initiated by the two interfaces to vacuum and silicon, respectively, which compete with each other during crystallization from the melt. Furthermore, the observation of the very different crystal morphologies in Figure 4i–l allows the assumption that the respective crystallization phenomena initiated at the two interfaces are very different and may have different kinetics. As such, the influence of kinetic factors during crystallization can be significant.

**Figure 5:**
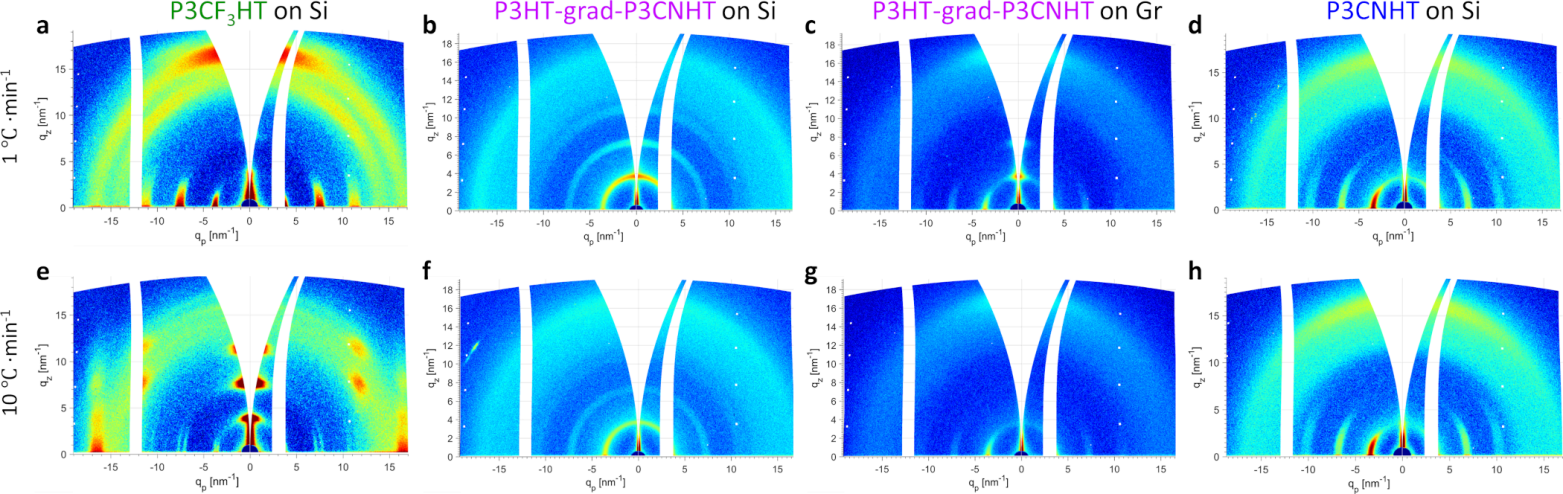
Influence of kinetics on crystal orientation of P3ATs films crystallized on silicon and graphene. GIWAXS patterns of (a, e) 85 nm thick P3CF_3_HT film on silicon, (b, f) 128 nm thick P3HT-grad-P3CNHT film on silicon, (c, g) 60 nm thick P3HT-grad-P3CNHT film on graphene, and (d) 95 nm and (h) 89 nm thick P3CNHT films on silicon measured at an incident angle of 0.18°, above the corresponding critical angles of the polymers, which are 0.163–165°. (a–d) GIWAXS patterns were obtained after crystallization during cooling from the melt at 1 °C·min^–1^, while (e–h) patterns were acquired after crystallization during cooling from the melt at 10 °C·min^–1^.

To test this assumption, we varied the cooling rate during crystallization from the melt in P3CF_3_HT and P3HT-grad-P3CNHT, another polymer with pronounced competition of interfacial interactions. Figure 5a,b,e,f displays the GIWAXS patterns of these two P3ATs crystallized on silicon at rates of 1 and 10 °C·min^–1^. As visible in Figure 5a,e, cooling the P3CF_3_HT film at 10 °C·min^–1^ suppresses the heterogeneous nucleation on silicon and, subsequently, promotes the crystallization at the vacuum interface, which results in an almost complete edge-on orientation. Unlike, the edge-on orientation formed in the P3HT-grad-P3CNHT film on silicon during slow cooling (Figure 5b) is suppressed during cooling at 10 °C·min^–1^ (Figure 5f). This effect can be observed even better for the P3HT-grad-P3CNHT film on graphene, as shown in Figure 5c,g. Here, the mixed edge-on and face-on crystal orientation formed during slow cooling (Figure 5c) was changed to a full face-on orientation by cooling the sample at 10 °C·min^–1^ (Figure 5g). Thus, these remarkable findings open a pathway to control the crystal orientation of the same P3AT on the same substrate just by varying the crystallization conditions given that the interfacial interactions at the substrate and vacuum interfaces compete during crystallization. In contrast, the crystal orientation in the P3CNHT film on silicon remains unaffected after fast cooling (Figure 5d,h), since the interface to vacuum has no impact on the crystallization of this polymer.

Finally, it remains to be seen whether the strategy applied to P3ATs can be extended to other CPs. As recently reported, the state-of-the-art CP, diketopyrrolopyrrole-based copolymer with two thiophene flanking units PDPP[T]_2_-T, is liquid crystalline at room temperature, unlike P3HT, and appears to have an even stronger preference for edge-on orientation after ordering from the melt on a silicon substrate than P3HT.^50^ This makes it a suitable candidate for the side-chain engineering strategy used in this work. Thus, we added an additional cyanohexyl side chain, equivalent to that in P3CNHT, to the thiophene flanking unit in PDPP[T]_2_-T to create a novel copolymer PDPP[T]_2_{2-HD}_2_-T{CNH} (Figures S16 and S17). Both polymers were spin-coated onto silicon and ordered during cooling from the melt. While PDPP[T]_2_-T on silicon was, as expected, exclusively edge-on oriented, the modified PDPP[T]_2_{2-HD}_2_-T{CNH} on silicon clearly showed a mixed edge-on and face-on crystal orientation with the apparent dominance of the face-on orientation (Figure S18). Although only one of the five side chain ends in its monomer carried the cyano group, it was sufficient to induce the face-on orientation in this CP, clearly demonstrating the validity of our approach.

## Conclusions

In this work, we showed that the crystal orientation formed in films of P3ATs can be significantly influenced and effectively tuned by side-chain engineering. Our approach is based on the theoretical prediction that the interface-induced crystallization is largely affected by the surface and interfacial energies. Thus, we purposely increased the surface energies at the vacuum interface in a series of P3ATs by chemically modifying the end group of the P3AT side chains. As a result, crystallization at the vacuum interface in P3AT with the most polar end group on the side chains, P3CNHT, was thermodynamically unfavorable and was completely suppressed. It allowed the oriented crystal growth initiated at the substrate interface to extend throughout the polymer film. In particular, we have shown that the uniform equilibrium face-on orientation required for effective vertical charge transport can be realized in films of P3CNHT on graphene as thick as 200 nm, which is a device-relevant dimension and, to our knowledge, has never been achieved before. Furthermore, the less specific silicon substrate, which is inactive for the crystallization of P3HT, was found to induce heterogeneous nucleation in the P3ATs with polar end groups on the side chains, resulting in a strong face-on orientation. This finding suggests an easier selection of substrates for oriented crystal growth in modified P3ATs. Furthermore, we have shown that if the competition of interfacial interactions exists in P3ATs films, the crystallization kinetics can be used to promote the crystallization phenomena at the lower or upper interface, leading to different dominant crystal orientations preferred for either horizontal or vertical charge transport. In this way, control of the crystal orientation can be achieved by a targeted manipulation of the crystallization conditions for the same sample. Finally, we showed that the proposed side-chain engineering strategy is successfully applied to induce face-on orientation in the state-of-the-art CP, polydiketopyrrolopyrrole. Given that the majority of CPs share the same backbone-side-chain architecture, we expect that our strategy and findings can be applied to control crystal orientation in films of many other CPs, thus, extending beyond the materials studied here.

## Supporting Information Available

Additional details on material synthesis, methods, and experiments, including NMR, MALDI-ToF, TGA, DSC, contact angle measurements, GIWAXS, AFM, and OM (PDF)
