# Supplementary material for: Controlling Crystal Orientation in Films of Conjugated Polymers by Tuning the Surface Energy
Source: Macromolecules. 2024 Oct 29;57(21):10399–409. doi: 10.1021/acs.macromol.4c01819 (PMC11562962; doi:10.1021/acs.macromol.4c01819)
Supplement: Supplementary file 1 — ma4c01819_si_001.pdf [file ma4c01819_si_001.pdf]

Supporting Information for

Controlling crystal orientation in films of conjugated polymers by tuning the surface energy

Oleksandr Dolynchuk,* Robert T. Kahl, Florian Meichsner, Alexander J. Much, Andrii Pechevystyi, Anna Averkova, Andreas Erhardt, Mukundan Thelakkat, Thomas Thurn-Albrecht

*Corresponding author: Oleksandr Dolynchuk
Email: oleksandr.dolynchuk@physik.uni-halle.de

This PDF file includes:

Supporting text
Figures S1 to S18
Table S1

Supporting Information Text

Methods

UV/Vis-spectroscopy. The UV/Vis spectra of the polymer films were recorded with a Jasco V-670 spectrophotometer. For solution measurements 0.02 mg/ml polymer solutions were prepared. Solutions of the polymer in a good solvent (THF) were measured. For the film spectra the polymers were spin coated from 2 mg/ml solutions in THF at 3000 rpm with for 60 seconds. The films were measured as cast and after crystallization from the melt. Melting of the films was realized by heating to a temperature above the respective melting temperature and holding under an argon atmosphere for 10 min.

Thermogravimetric Analysis (TGA). TGA thermograms were recorded on a Mettler Toledo TGA/DSC 3. Samples (ca. 10-20 mg) were placed in aluminum oxide ceramic crucibles (70 µL) and heated from 30 to 500°C at a rate of 10°C·min^{-1} under nitrogen atmosphere. The temperature was increased in 10°C steps and kept constant for 60 min before going to the next temperature step.

Differential scanning calorimetry (DSC). DSC measurements were performed with a power-compensated DSC7 and DSC 8000 from PerkinElmer equipped with PerkinElmer Intracooler 2 for controlled cooling and heating. Samples were sealed in 20 mL aluminum pans. Heat-flow rate data were obtained during heating and cooling the samples from -50 to 160°C (P3BrHT) and 260°C (P3HT, P3CF$_3$HT, P3CNHT, P3HT-grad-P3CNHT) at a rate of 10°C/min. The raw heat-flow rate data were corrected for the instrumental asymmetry and converted into the temperature dependencies of the apparent specific heat capacity.

Surface energy determination of SAMs. Sessile drop contact angle measurements on self-assembled monolayers (SAMs) of three different silanes (Trimethoxypropylsilane, trimethoxy(3-bromopropyl)silane, trimethoxy(3,3,3-trifluoropropyl)silane and trimethoxy(3-cyanopropyl)silane) simulating the ω-side chain functionalization were conducted at 20°C with water, ethylene glycol and diiodomethane. Trimethoxypropylsilane, trimethoxy(3-bromopropyl)silane and trimethoxy(3,3,3-trifluoropropyl)silane were purchased from TCI and trimethoxy(3-cyanopropyl)silane was purchased from abcr GmbH. The surface energy of the SAMs was determined by the Owens, Wendt, Rabel and Kaelble (OWRK) method (1,2). Furthermore, a determination of the surface energy according to Neumann was conducted from the contact angle measurements with water (2,3).

Synthesis and Sample Preparation

Synthesis of 2,5-Dibromo-3-(6-bromohexyl)thiophene. 2,5-Dibromo-3-(6-bromohexyl)thiophene was synthesized according to literature (4).

Synthesis of 3-(6-trifluorohexyl)thiophene - 3-FHT. Mg (22.4 mmol, 1.1 eq.) and ether were added to a dry Schlenk flask and activated with a few drops of $Br_2C_2H_2$. Afterwards 1/10th of the 1,1,1-trifluoro-6-bromohexane (20.5 mmol, 1 eq.) was added dropwise before the residual alkylhalide dissolved in ether (ca. 5 ml) was added slowly. The reaction solution was allowed to stir for 3 h. In a separate Schlenk flask, 3-bromothiophene (20.5 mmol, 1 eq.) and Ni(dppp)Cl_2 (0.205 mmol, 0.01 eq.) were dissolved in diethyl ether. After complete dispersion of Ni(dppp)Cl_2 the Grignard reagent was injected slowly, while cooling with an ice bath, and the reaction mixture was allowed to stir over night at 50 °C before it was quenched with ice and 1 M HCl. The mixture was extracted with diethyl ether and the organic phases were combined and dried over Na_2SO_4 before solvent was evaporated and the product was purified by vacuum distillation (1.6 x 10^{-1} mbar, T_v = 40 °C).

1H-NMR: δ_H (300 MHz; ppm, $CDCl_3$): 7.27-7.24 (m, 1 H), 6.96-6.93 (m, 2 H), 2.7-2.62 (t, 2 H), 2.17-1.99 (m, 2 H), 1.73-1.53 (m, 4 H), 1.48-1.30 (m, 2H).

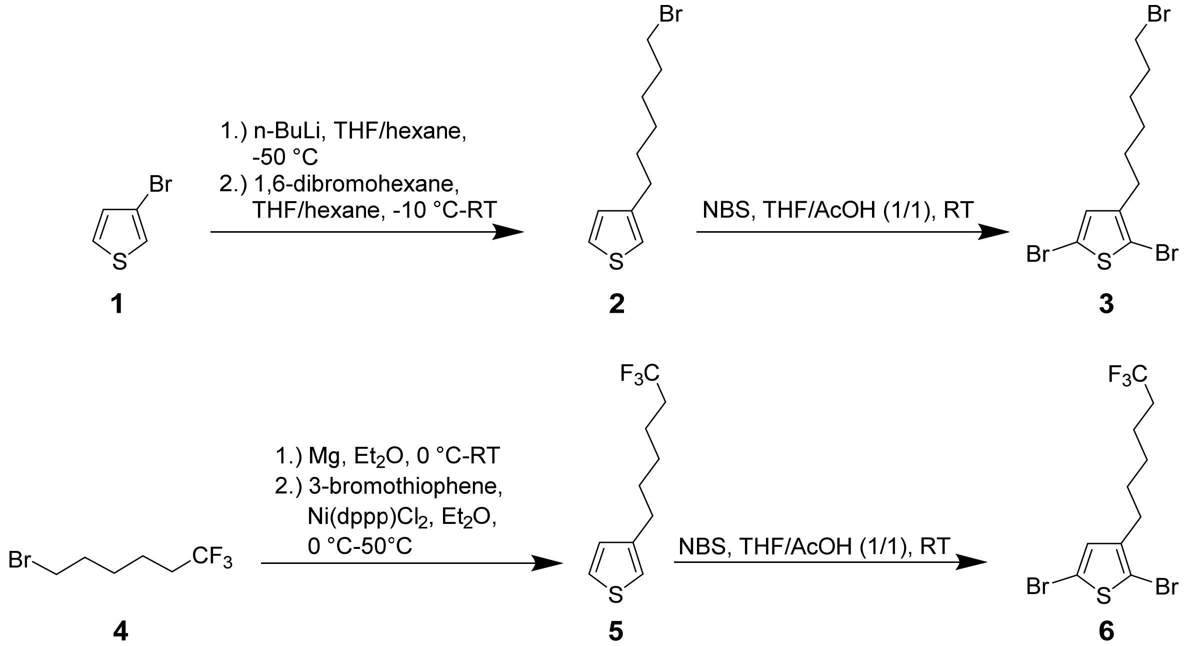

Synthesis of 2,5-Dibromo-3-(6-trifluorohexyl)thiophene – 2,5-DiBr-3-FHT. 3-(6,6,6-trifluorohexyl)thiophene (10 mmol, 1 eq.) was dissolved in a mixture of THF/acetic acid (v:v 1:1, 11 ml) under the exclusion of light. Subsequently NBS (23 mmol, 2.3 eq.) was added to the solution and the solution was stirred for 2 h. The reaction mixture was extracted with ethyl acetate against water, sodium bicarbonate and NaOH, the organic phase was collected, dried over Na_2SO_4 and the solvent is removed under reduced pressure. The crude product was purified by column chromatography (silica, eluent hexane).

1H-NMR: δ_H (300 MHz; ppm, $CDCl_3$): 6.8 (s, 1 H), 2.59-2.50 (t, 2 H), 2.18-1.98 (m, 2 H), 1.67-1.53 (m, 4 H), 1.47-1.33 (m, 2 H).

Synthesis of Poly-(3-(6-bromohexyl))thiophene - P3BrHT. 2,5-Dibromo-3-(6-bromohexyl)thiophene (7.523 mmol, 1 eq.) was dried in high vacuum for 30 min and dissolved in THF (15 ml) at a concentration of 0.5 M. Subsequently *t*-BuMgCl (1.416 M in THF, 7.222 mmol, 0.96 eq.) was added and the reaction mixture was allowed to stir for 20 h under the exclusion of light. The reaction mixture was diluted with THF (60 ml) to 0.1 M before a dispersion of Ni(dppp)Cl_2 (0.075 mmol, 0.01 eq.) in 2 ml THF was added in one shot to start polymerization. After 2 h the polymerization was quenched with 1 M HCl and the polymer was precipitated in MeOH. The polymer was purified by Soxhlet extraction with MeOH and hexane and dried in vacuum (46).

1H-NMR: δ_H (300 MHz; ppm, $CDCl_3$): 7.00 (s, 1 H), 3.5-3.35 (t, 2 H), 2.92-2.72 (t, 2 H), 2.00-1.81 (m, 2 H), 1.8-1.64 (m, 2 H), 1.58-1.38 (m, 4 H).

SEC: M_n = 12,100 g/mol, $Đ$ = 1.35.

MALDI-ToF: 12,300 g/mol

Synthesis of Poly-(3-(6-cyanohexyl))thiophene - P3CNHT. P3BrHT (300 mg, 1 eq.) was dissolved in THF/DMSO (v/v: 4/1) and KCN (632 mg, 8 eq. per r.u.) was added. The reaction mixture then was stirred under reflux (80 °C) for 12 h. The polymer was precipitated in MeOH and washed with $CHCl_3$ in a Soxhlet apparatus to ensure complete removal of remaining KCN. The polymer was dried in vacuum.

1H-NMR: δ_H (300 MHz; ppm, $CDCl_3$): 7.00 (s, 1 H), 2.92-2.72 (t, 2 H), 2.4-2.3 (t, 2 H), 1.87-1.6 (m, 4 H), 1.57-1.37 (m, 4 H).

IR: 2244 cm^{-1} C≡N

SEC: M_n = 10,100 g/mol, $Đ$ = 1.35.

MALDI-ToF: 11,100 g/mol

Synthesis of Poly-(3-(6-trifluorohexyl))thiophene - P3CF$_3$HT. In a dry Schlenk flasks the 2,5-dibromo-3FHT (3.98 mmol, 1 eq.) was dissolved in dry THF (8 ml) and *t*-BuMgCl (3.821 mmol, 0.96 eq.) was added. After complete Grignard monomer formation (5 h), the reaction solution was diluted with dry THF (32 ml) and the polymerization was initiated by the addition of a dispersion of Ni(dppp)Cl_2 (0.0398 mmol, 0.01 eq.) in 2 ml THF to the reaction mixture in one shot. The polymerization was quenched with HCl, the solution was concentrated and precipitated in methanol. The polymer is dried in vacuum and purified by Soxhlet extraction with MeOH and hexane.
1H-NMR: δ_H (300 MHz; ppm, $CDCl_3$): 6.99 (s, 1 H), 3.00-2.74 (t, 2 H), 2.21-1.99 (t, 2 H), 1.82-1.58 (m, 4 H), 1.57-1.31 (m, 2 H).
SEC: M_n = 22,000 g/mol, $Đ$ = 1.26.
MALDI-ToF: 13,300 g/mol
Synthesis of Poly-3-hexylthiophene-gradient-Poly-(3-(6-bromohexyl))thiophene - P3HT-grad-P3BrHT. In a dry Schlenk flasks the 2,5-dibromo-3-HT (3.107mmol, 0.5 eq) and 2,5-dibromo-3-BrHT (3.047 mmol, 0.5 eq.) were added and dissolved in dry THF (12 ml, 0.5 M) before t-BuMgCl (5.908 mmol, 0.96 eq.) was added. After complete Grignard monomer formation (4 h), the reaction solution was diluted with dry THF (49 ml, 0.1 M) and the polymerization was initiated by the addition of a dispersion of Ni(dppp)Cl_2 (0.061 mmol, 0.01 eq.) in 2 ml THF to the reaction mixture in one shot. The polymerization was allowed to run for 90 min. The polymerization was quenched with HCl, the solution was concentrated and precipitated in methanol. The polymer was dried in vacuum and purified by Soxhlet extraction with MeOH and hexane.
1H-NMR: δ_H (300 MHz; ppm, $CDCl_3$): 7.00 (s, 1 H), 3.50-3.40 (t, 2 H), 2.98-2.65 (t, 4 H), , 1.84-1.66 (m, 2 H), 1.65-1.23 (m, 10 H), 1.00-0.82 (t, 3 H).
SEC: M_n = 21,600 g/mol, $Đ$ = 1.18.
MALDI-ToF: -
Synthesis of Poly-3-hexylthiophene-gradient-Poly-(3-(6-cyanohexyl))thiophene - P3HT-grad-P3CNHT. P3HT-grad-P3BrHT (400 mg, 1 eq.) was dissolved in THF (16 ml) and DMSO (4 ml) before KCN (870 mg, 8 eq. per. r.u.) in was added to the solution which then was heated under reflux and stirred overnight. The product was precipitated in methanol and purified by Soxhlet extraction with $CHCl_3$ to ensure complete removal of residual KCN. The polymer was precipitated in MeOH and dried in the vacuum.
1H-NMR: δ_H (300 MHz; ppm, $CDCl_3$): 7.00 (s, 1 H), 2.98-2.65 (t, 4 H), 2.44-2.30 (t, 2 H), 1.84-1.66 (m, 2 H), 1.65-1.23 (m, 10 H), 1.00-0.82 (t, 3 H).
SEC: M_n = 23,600 g/mol, $Đ$ = 1.28.
MALDI-ToF: -
Preparation of silane monolayers with varying end groups for surface energy measurements. Glass substrates were cleaned by ultransonification in Hellmannex (2 %), water, acetone and isopropyl alcohol for 5 minutes each, before they were treated with ozone at 50°C for 15 min. The ozone treated substrates were immersed into 1 wt% solutions of the respective silane (Trimethoxypropylsilane, trimethoxy(3-bromopropyl)silane, trimethoxy(3,3,3-trifluoropropyl)silane and trimethoxy(3-cyanopropyl)silane) for 60 min at 100°C (Fig. S7). Afterwards the functionalized substrates were rinsed with toluene and isopropyl alcohol and dried in the nitrogen jet.
Synthesis of PDPP[T]$_2${2-HD}$_2$-T{CNH}. For the synthesis of PDPP[T]$_2${2-HD}$_2$-T{CNH}, first the brominated precursor polymer PDPP[T]$_2${2-HD}$_2$-T{BrH} was synthesized, followed by polymer analogous nucleophilic substitution on the brominated sites.
Stille Polycondensation – PDPP[T]$_2${2-HD}$_2$-T{BrH}. Polymerization reactions were conducted according to Müller et al., with 3,6-bis(5-bromothiophen-2-yl)-2,5-bis(2-hexyldecyl)-2,5-dihydropyrrolo[3,4-c]pyrrole-1,4-dione (DPP{HD}$_2$[T]$_2$$Br_2$) and 3-(6-bromohexyl)thiophene-2,5-diyl)bis(trimethylstannane) (BrHT($SnMe_3$)$_2$) as monomers, followed by Soxhlet removal of low molecular mass polymers (5). The purified product was extracted with chloroform to obtain a polymer with an average molecular mass of M_n = 15,100 g mol^{-1} and a dispersity of $Đ$ = 1.8 (cf. Fig. S16).
Polymer analogous Substitution – PDPP[T]$_2${2-HD}$_2$-T{CNH}. The conversion of PDPP-BrHT to PDPP-CNHT was conducted based on the substitution of P3BrHT to P3CNHT: 100 mg of PDPP-

BrHT was dissolved in a mixture of chlorobenzene and DMSO (v : v = 4 : 1), followed by the addition of 52 mg KCN (0.8 mmol, 8 eq. relative to the polymer repeating unit). The mixture was heated to 60 °C and reacted for three days. After cooling to room temperature, water was added and the polymer was carefully extracted with chloroform. The organic phase was washed three times with water to remove residual KCN and KBr. After purification, 94 mg (100 %) of dark green polymer was obtained. Full conversion of the terminal side chain functionalities is evident, based on a high-field NMR shift of the neighboring protons in ^{1}H-NMR spectra (Fig. S15). Further, GPC analysis (Fig. S16) hints at a subtle increase in molecular mass M_n = 17,700 g mol^{-1}, $Đ$ = 1.8, which is likely caused by a changed hydrodynamic radius of the polymer.

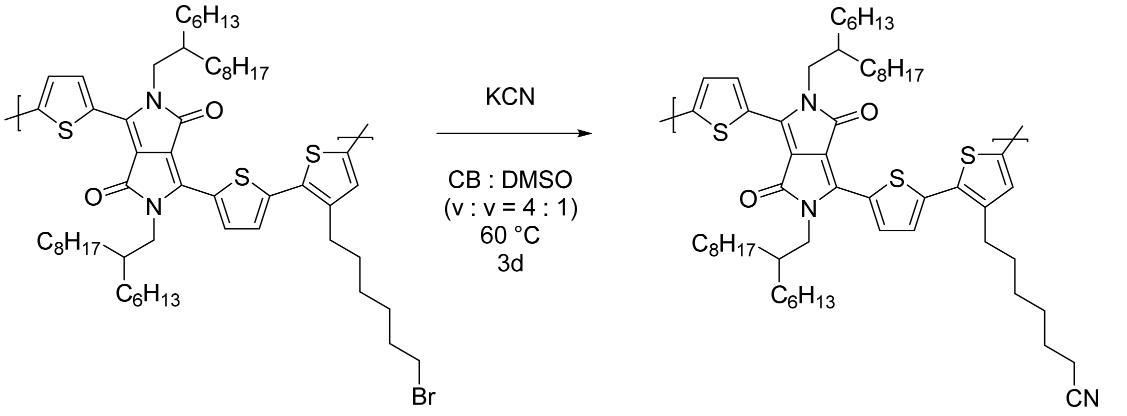

PDPP[T]$_2$-T. PDPP[T]$_2$-T is a commercially available sample purchased from Ossila (Sheffield, England) and was used as received. The molar mass M_n = 45,400 g/mol and dispersity $Đ$ = 2.94 of the sample were determined by GPC with polystyrene calibration and THF as eluent.

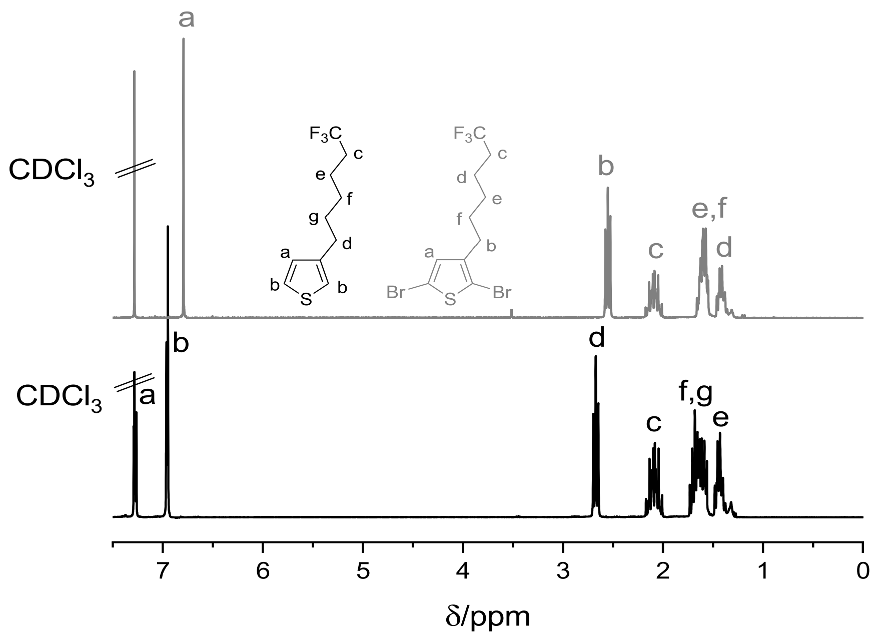

Fig. S1. 1H-NMR of the new 3-CFHT and 2,5-DiBr-3-CFHT measured in $CDCl_3$.

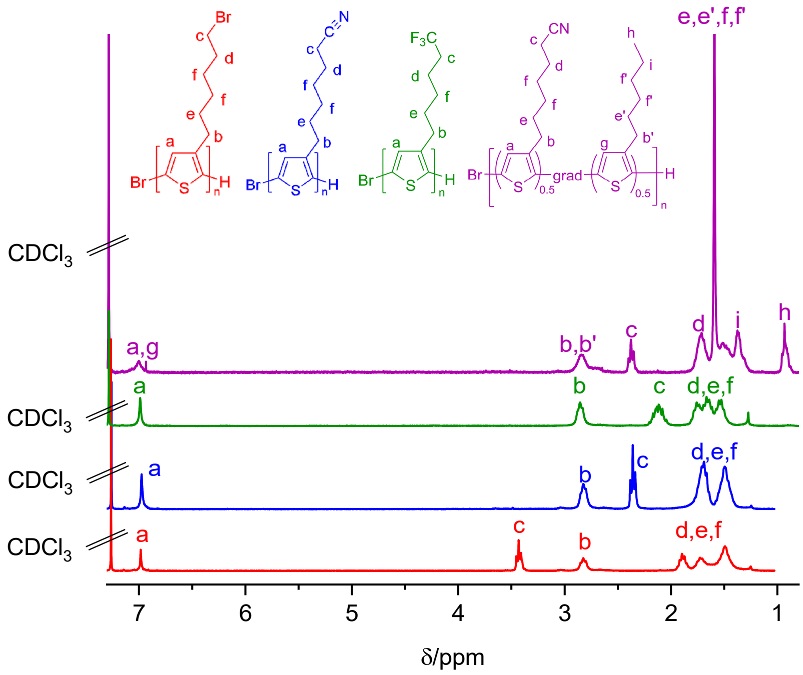

Fig. S2. 1H-NMR of P3BrHT, P3CF$_3$HT, P3CNHT and P3HT-grad-P3CNHT measured in CDCl$_3$. The protons c in P3BrHT shift from δ= 3.47 ppm to δ= 2.35 ppm (for the protons c') in P3CNHT. The protons d in P3BrHT also show a shift to the high field after the conversion to P3CNHT: δ_d= 1.9 ppm in P3BrHT to $\delta_{d'}$=1.75 ppm in P3CNHT. For P3CF$_3$HT, the signal of the protons c'' in direct vicinity to the CF$_3$-group arise at chemical shift δ= 2.2 ppm, whereas the protons d'' show a chemical shift of δ= 1.76 ppm.

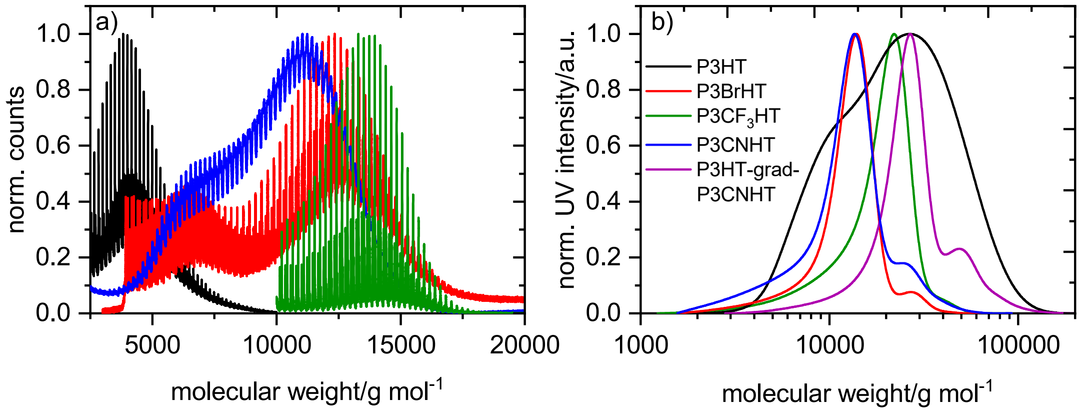

Fig. S3. a), MALDI-ToF spectra of P3HT (black), P3BrHT (red), $P3CF_3HT$ (green) and P3CNHT (blue). Note: Here for P3HT due to the high dispersity difficulties in the measurement arose and only the low molecular weight fraction was flying. For the gradient copolymer no MALDI could be obtained because the copolymer was not flying. **b)**, SEC traces for P3HT, P3BrHT, $P3CF_3HT$, P3CNHT and P3HT-grad-P3CNHT (eluent THF).

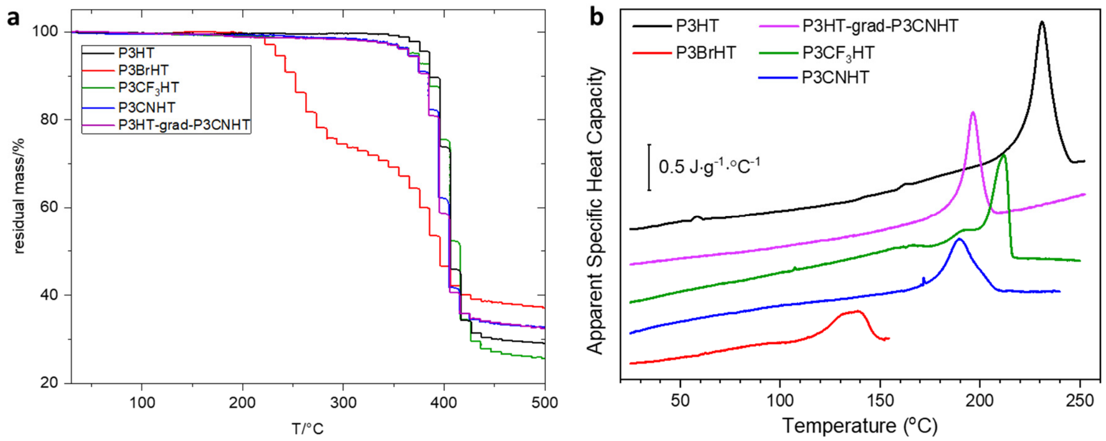

Fig. S4. (**a**), TGA curves from isothermal measurements for P3HT, P3BrHT, $P3CF_3HT$, P3CNHT and P3HT-grad-P3CNHT. The temperature was increased in 10 °C steps and kept constant for 60 min before going to the next temperature step. (**b**), DSC heating curves of P3HT, P3BrHT, P3CF3HT, P3CNHT and P3HT-grad-P3CNHT measured at a heating rate of 10 $°C·min^{-1}$ after crystallization from the melt during cooling at a rate of 10 $°C·min^{-1}$.

Table S1. Crystal symmetry and unit cell parameters for the investigated P3ATs. The data for P3HT and P3BrHT were reported in our previous publication (4). The determination of the unit cell parameters was performed based on the scattering patterns of bulk samples with isotropic crystal orientation as well as thin films crystallized on graphene and silicon with face-on and/or edge-on crystal orientation.

**Polymers**	**P3HT**	**P3BrHT**	**P3HT-grad-P3CNHT**	**P3CF_3HT**	**P3CNHT**
**Crystal symmetry**	monoclinic	triclinic	monoclinic	triclinic	monoclinic
**a** (nm)	1.677	1.778	1.779	1.733	1.817
**b** (nm)	0.757	0.773	0.759	0.786	0.753
**c** (nm)	0.788	1.153	0.788	0.793	0.789
**α** (°)	90	91.1	90	91.6	90
**β** (°)	90	91.1	90	90.4	90
**γ** (°)	92.7	91.8	~93	~90	91.8

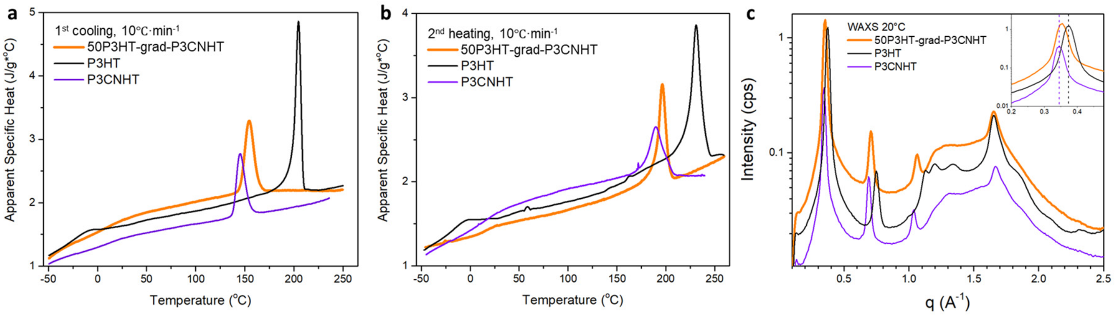

Fig. S5. DSC cooling (**a**) and heating (**b**) curves as well as WAXS patterns (**c**) at 20 °C of P3HT, P3CNHT, and P3HT-grad-P3CNHT. The crystallization and melting of P3HT-grad-P3CNHT in DSC show single peaks with temperatures intermediate between those of P3HT and P3CNHT. In the WAXS, P3HT-grad-P3CNHT shows one crystal structure with the position of (100) peak being in-between of the (100) peaks of P3HT and P3CNHT. These findings clearly prove that the 3HT and 3CNHT monomers in P3HT-grad-P3CNHT cocrystallize and build a common crystal lattice.

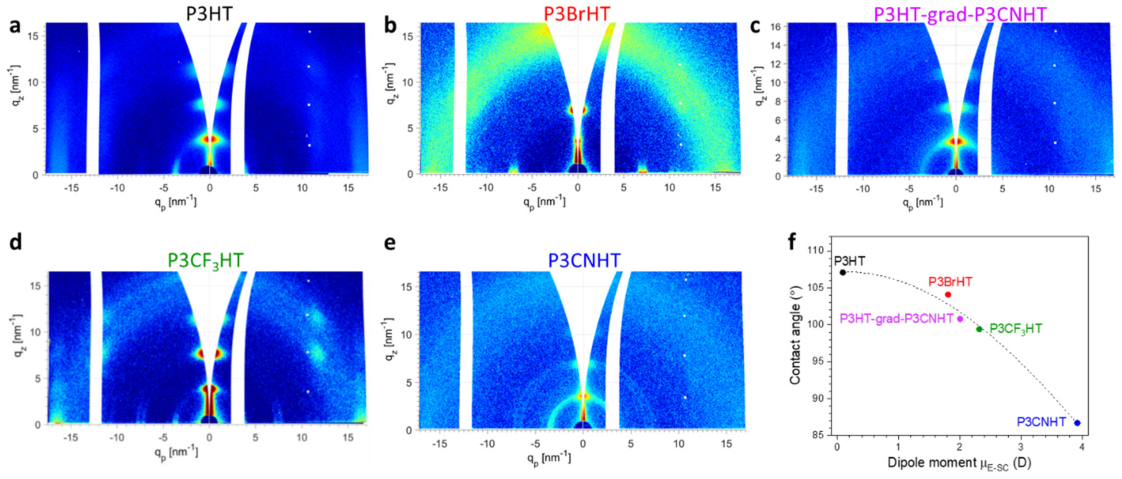

Fig. S6. T (**a**-**e**), GIWAXS patterns of 31 nm thin P3HT (**a**), 26 nm thin P3BrHT (**b**), 28 nm thin P3HT-grad-P3CNHT (**c**), 29 nm thin P3CF$_3$HT (**d**), and 23 nm thin P3CNHT (**e**) on silicon after crystallization in the vacuum oven (**a**,**b**,**e**) or on the Linkam hot stage (**c**,**d**). As previously reported in our publication, edge-on crystal orientation is the dominant orientation observed in the top layer of P3HT and P3BrHT films (7). The nearly complete edge-on crystal orientation is also evident in other P3AT films. It is important to note that the weak isotropic part of the (100) intensity in (**e**) is split and can be securely attributed to the signal originating from the edges of the sample. Since the dominant (100) reflection is located on the meridian, the crystal orientation in the 23 nm thin P3CNHT film on silicon is edge-on. Therefore, our results demonstrate that the top layer of all P3ATs on silicon exhibits the same dominant edge-on crystal orientation. (**f**), The water droplet contact angle formed on P3AT films on silicon, for which the GIWAXS patterns are shown in (**a**-**e**), as a function of the dipole moment of small molecules $\boldsymbol{\mu_{E-SC}}$ equivalent to the end group of P3AT side chains – ethane for P3HT, methyl bromide for P3BrHT, 1,1,1-trifluoroethane for P3CF3HT, and acetonitrile for P3CNHT (6). The value of $\boldsymbol{\mu_{E-SC}}$ in the case of P3HT-grad-P3CNHT was estimated as the average of $\boldsymbol{\mu_{E-SC}}$ for ethane and acetonitrile.

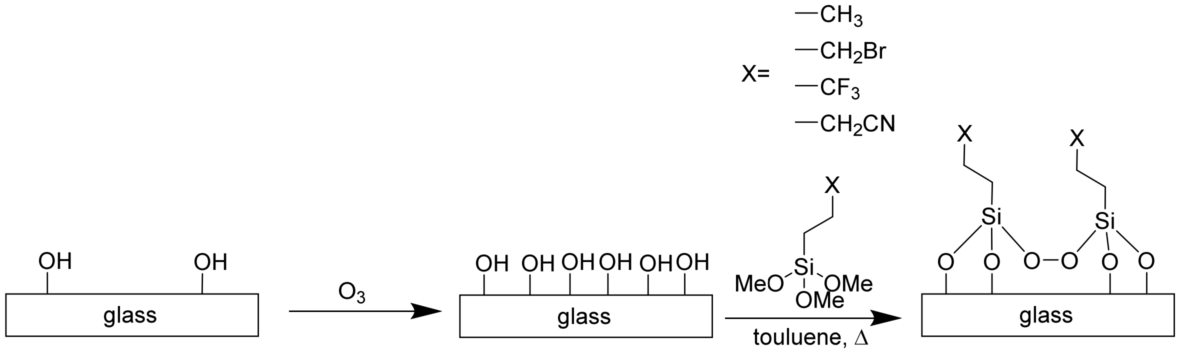

Fig. S7. Synthetic scheme of the preparation of the different SAMs simulating the ω-side chain functionalization of the polymers.

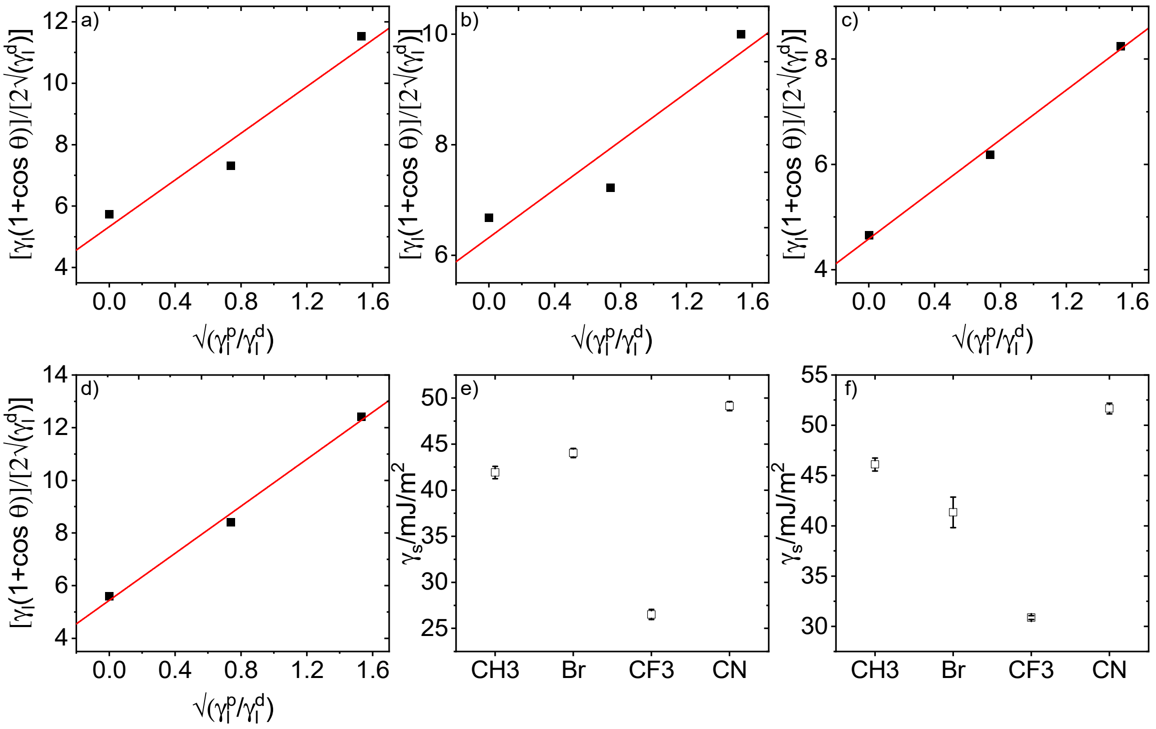

Fig. S8. Linear regression for the determination of the polar and disperse contributions of the surface energy of SAMs carrying CH_3-head groups (**a**), Br-head groups (**b**), CF_3-head groups (**c**) and CN-head groups (**d**). The surface energy of the SAM carrying the respective end group determined by the OWRK (**e**) (1,2) and the Neumann (2,3) methods from the contact angle measurements with water (**f**).

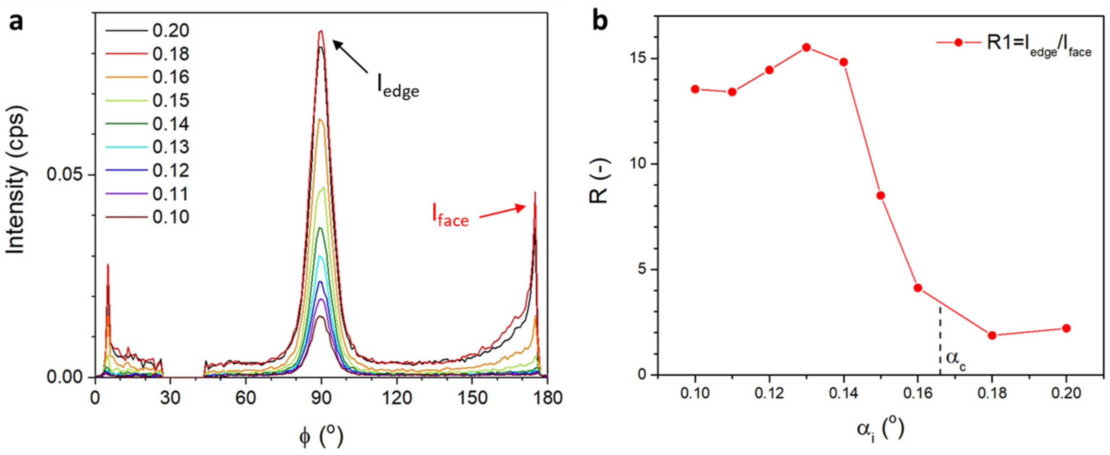

Fig. S9. (**a**), The azimuthal distribution of intensity of the (100) reflection of 60 nm thick P3HT-grad-P3CNHT film on graphene measured under various angles of incidence indicated in the figure legend. The azimuthal distributions of intensity were extracted directly from the measured scattering patterns (detector images) by integrating the intensity over a small $\boldsymbol{q}$-range around the (100) reflection. Note that the dependencies shown are not corrected for the curvature of the Ewald sphere. (**b**), Ratio of intensities I_{edge}/I_{face} of the (100) reflection of 60 nm thick P3HT-grad-P3CNHT film on graphene scattered from edge-on and face-on crystals as a function of incident angle α_i. The large increase of I_{edge}/I_{face} with decreasing α_i evidences that edge-on crystals dominate in the top layer of the film and, thus, are induced by the interface to vacuum, while face-on crystals are induced by the interface to graphene.

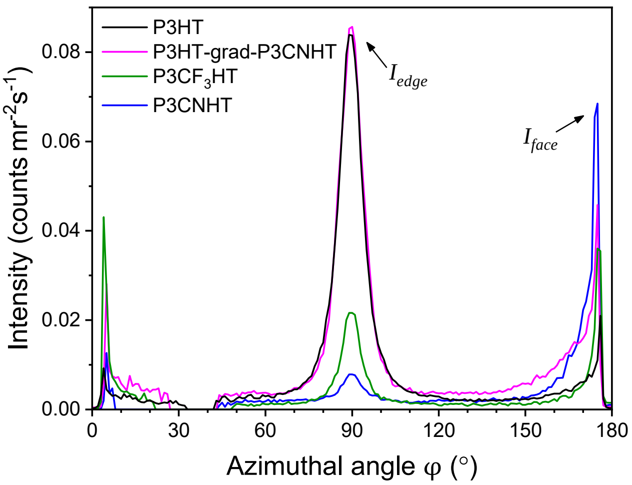

Fig. S10. The azimuthal distribution of intensity of the (100) reflection of 46 nm thick P3HT, 60 nm thick P3HT-grad-P3CNHT, 55 nm thick $P3CF_3HT$, and 44 nm thick P3CNHT on graphene, whose respective GIWAXS patterns are shown in Fig. 3 in the main text. The azimuthal distributions of intensity were extracted directly from the measured scattering patterns (detector images) by integrating the intensity over a small $\boldsymbol{q}$-range around the (100) reflection. Note that the dependencies shown are not corrected for the curvature of the Ewald sphere. Thus, the intensity values taken at φ = 90° are slightly off the direction along $\boldsymbol{q_z}$ in the reciprocal space, as can be seen in Fig. 3 in the main text. However, such a small error only marginally influences the quantitative dependencies in Fig. 3h in the main text and does not affect the qualitative conclusions drawn from them. The arrows in the figure indicate the intensity scattered from edge-on and face-on oriented crystals, which were used to compute the ratio $I^{edge}_{(100)}/I^{face}_{(100)}$. The Hermans orientation parameter was calculated from the region of the curves within the range of $90° < \varphi < 180°$, which was not affected by the detector blank area. For the calculation of the Hermans orientation parameter, the direction to the meridian (φ = 90°) was set to 0° and the direction to the equator (φ = 180°) – to 90°, as is usual for such calculations.

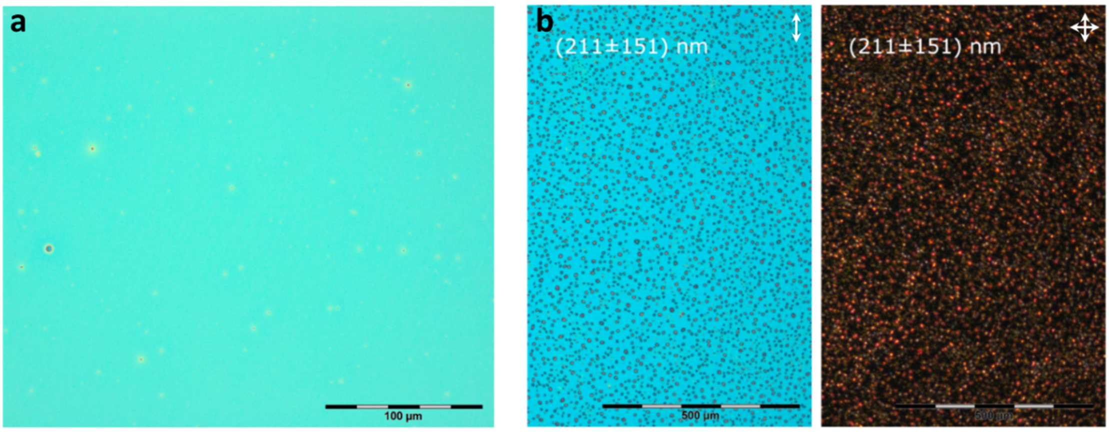

Fig. S11. The optical microscopy images of 55 nm thick P3CF$_3$HT on graphene after spin coating (**a**) and after crystallization from the melt (**b**). The image (**b**) shows the same area of the film taken with open (left half of the image) and crossed (right half of the image) polarizers. The mean droplet height indicated in the image (**b**) was determined with a profilometer.

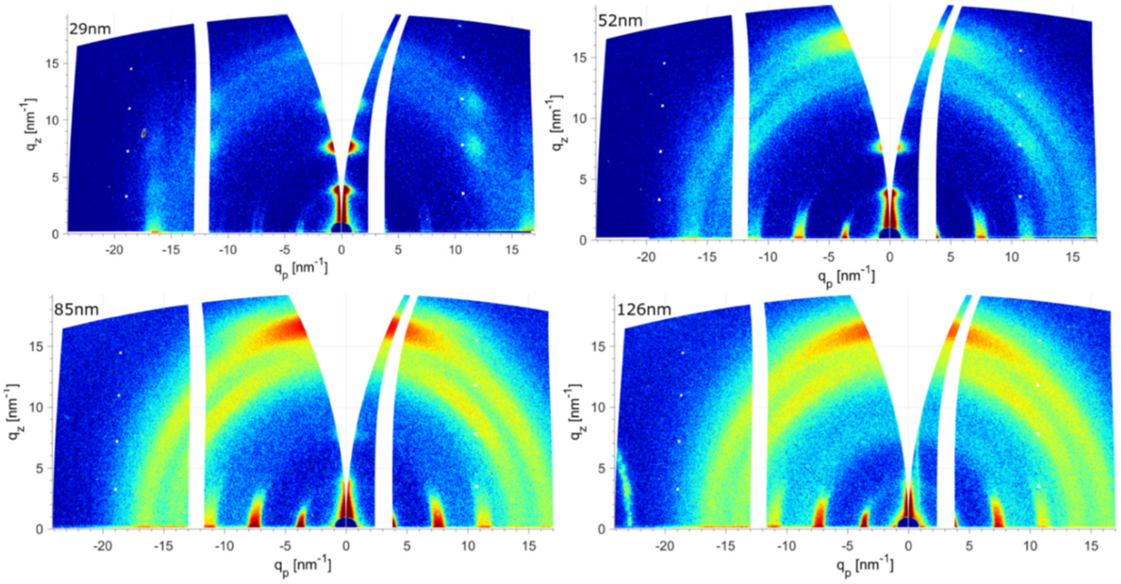

Fig. S12. The GIWAXS patterns of P3CF$_3$HT films with different thicknesses on silicon crystallized from the melt during cooling at 1 °C·min^{-1}. The film thickness is indicated in the upper left corner of each GIWAXS pattern. The results evidence a gradual change of the crystal orientation in P3CF$_3$HT with increasing film thickness from almost complete edge-on orientation for 29 nm thick film to complete face-on orientation for 126 nm thick film.

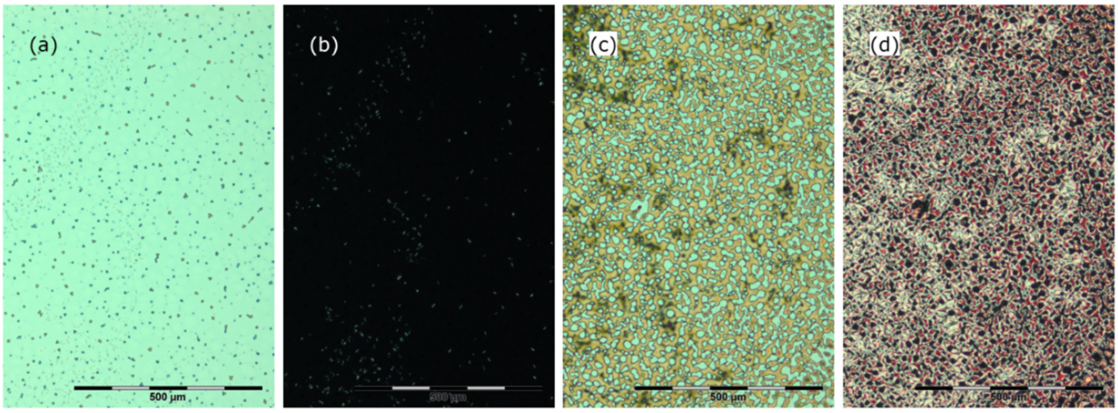

Fig. S13. The optical misroscopy images of P3CF$_3$HT films on silicon with thicknesses of 29 nm (**a**,**b**) and 126 nm (**c**,**d**) crystallized from the melt during cooling at 1 °C·min^{-1}. The images (**a**) and (**b**), as well as (**c**) and (**d**) show the same area of the respective films taken with open (**a**,**c**) and crossed (**b**,**d**) polarizers. While the thin film remains continuous after crystallization from the melt and shows only small birefringent inclusions, the thicker films partially dewets and displays strong birefringence caused by the spherulitic structures.

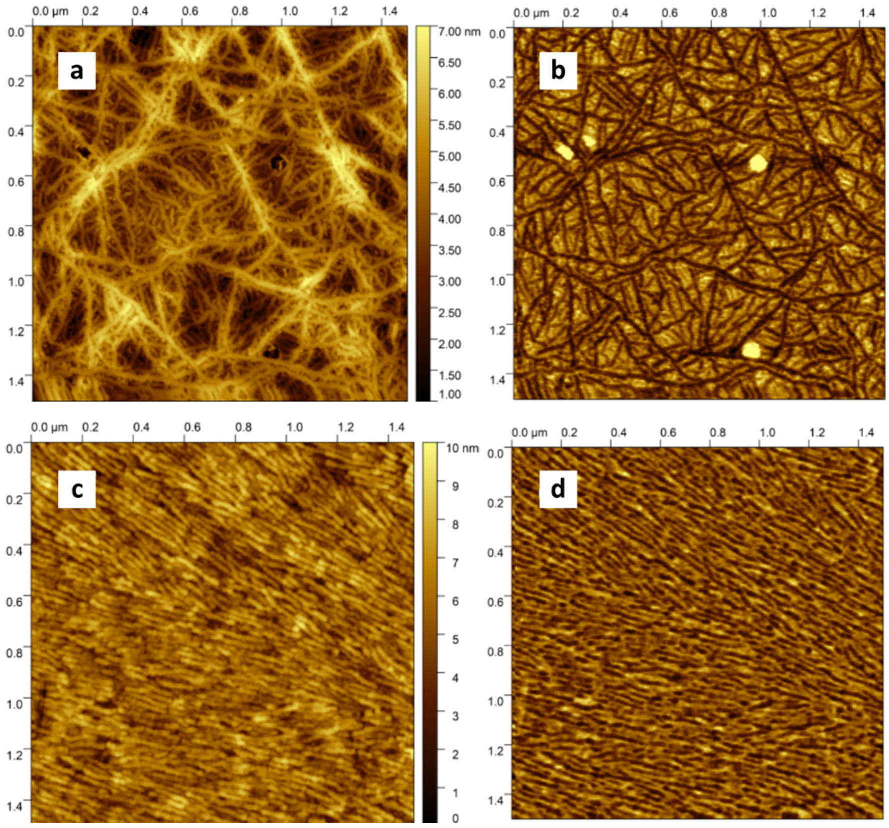

Fig. S14. The AFM height (**a**,**c**) and adhesion (**b**,**d**) images of 29 nm thick (**a**,**b**) and 126 nm thick (**c**,**d**) P3CF$_3$HT films on silicon crystallized from the melt during cooling at 1 °C·min^{-1}. The comparison of Figs. S11-13 allows concluding that edge-on crystals in P3CF$_3$HT are weakly birefringent and form a nanofibril lamellar morphology, whereas face-on crystals in P3CF$_3$HT organize into spherulitic structures with a stacked lamellar morphology.

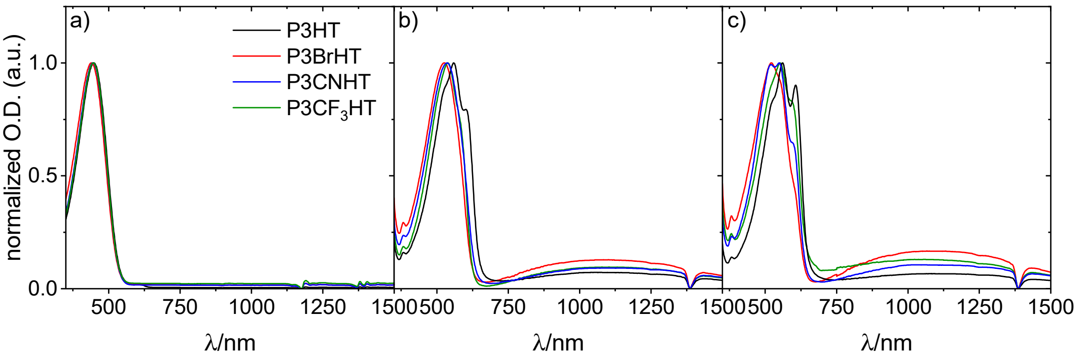

Fig. S15. (**a**), UV/VIS spectra of the polymers measured in THF (c = 0.02 mg/ml). (**b**), UV/VIS spectra of thin films of the polymer spincoated from THF solutions, measured as cast. (**c**), UV/VIS spectra of the polymer thin films spincoated from THF solutions, measured after melt crystallization.

For basic investigations of the aggregation behavior of the different polymers, UV/VIS studies were conducted. The solution spectra of the polymers in a good solvent (here: THF) showed no aggregation for any of the polymers but only a strong π-π*-absorption due to the individual polymer chains random coil conformation at λ_{max} = 440-450 nm for all homopolymers (Fig. S15a). In the thin film studies of as cast films of the polymers, a strong bathochromic shift of the π-π*-absorption for all polymers is visible ($\lambda_{\pi\text{-}\pi^*}$ = 525-535 nm). For P3HT $\lambda_{\pi\text{-}\pi^*}$ = 526 nm, furthermore two additional peaks at λ_1 = 559 nm and λ_2 = 602 nm arise (Fig. S15b). The two new peaks can be assigned to the 0-1 and 0-0 transition, indicating the presence H-aggregates.
In the case of the as-cast P3HT film, the ratio $A_{0\text{-}0}/A_{0\text{-}1}$ = 0.795, which indicates the presence of partially ordered H-aggregates. For P3BrHT, P3CF$_3$HT and P3CNHT however no additional vibronic features are visible in the spectra of the as cast films. At lower energies however for all polymers a broad absorption with a weak absorption is visible. A broad absorption in the range from 750-1500 nm usually originates from polaron formation on the polymer chains (Fig. S15b). The polaron formation could be explained by the oxidation of the polymers due to atmospheric oxygen. After crystallization from the melt of the polymer thin films, for P3BrHT, P3CF$_3$HT and P3CNHT also new vibronic features are arising (Fig. S15c). For P3HT, the $A_{0\text{-}0}$- and $A_{0\text{-}1}$-transition peak ($\lambda_{0\text{-}0}$= 607 nm, $\lambda_{0\text{-}1}$= 559 nm) become more pronounced, indicating a stronger aggregation and more ordered aggregates. For P3BrHT, the main absorption $\lambda_{\pi\text{-}\pi^*}$= 518 nm is still very pronounced, furthermore two vibronic features, which can be assigned to the $A_{0\text{-}0}$ - and $A_{0\text{-}1}$ -transition are arising ($\lambda_{0\text{-}0}$= 605 nm, $\lambda_{0\text{-}1}$= 559 nm), however they are way less pronounced than in P3HT, indicating less ordered aggregates in P3BrHT (Fig. S15c). For the P3CF$_3$HT spectra, the π-π*-absorption $\lambda_{\pi\text{-}\pi^*}$= 515 nm is visible as a weak shoulder of the very pronounced peak of the $A_{0\text{-}1}$-transition ($\lambda_{0\text{-}1}$= 554 nm), a second peak which can be assigned to the $A_{0\text{-}0}$-transition ($\lambda_{0\text{-}0}$= 596 nm) is arising (Fig. S14c). In comparison to P3HT, the aggregates are also less ordered, since the $A_{0\text{-}0}$-peak is less pronounced in P3CF$_3$HT. For the P3CNHT spectra, the π-π*-absorption $\lambda_{\pi\text{-}\pi^*}$= 517 nm is still as pronounced as the new arising $A_{0\text{-}1}$ -transition ($\lambda_{0\text{-}1}$= 550 nm), the second vibrionic band $\lambda_{0\text{-}1}$= 601 nm can be assigned to the $A_{0\text{-}0}$ -transition. The vibronic band of the 0-0-transition is way less pronounced than in P3HT and P3CF$_3$HT indicating less ordered aggregates. In comparison with P3BrHT, however, the aggregates in P3CNHT are more ordered, since the ratio $A_{0\text{-}0}/A_{0\text{-}1}$ is increased.

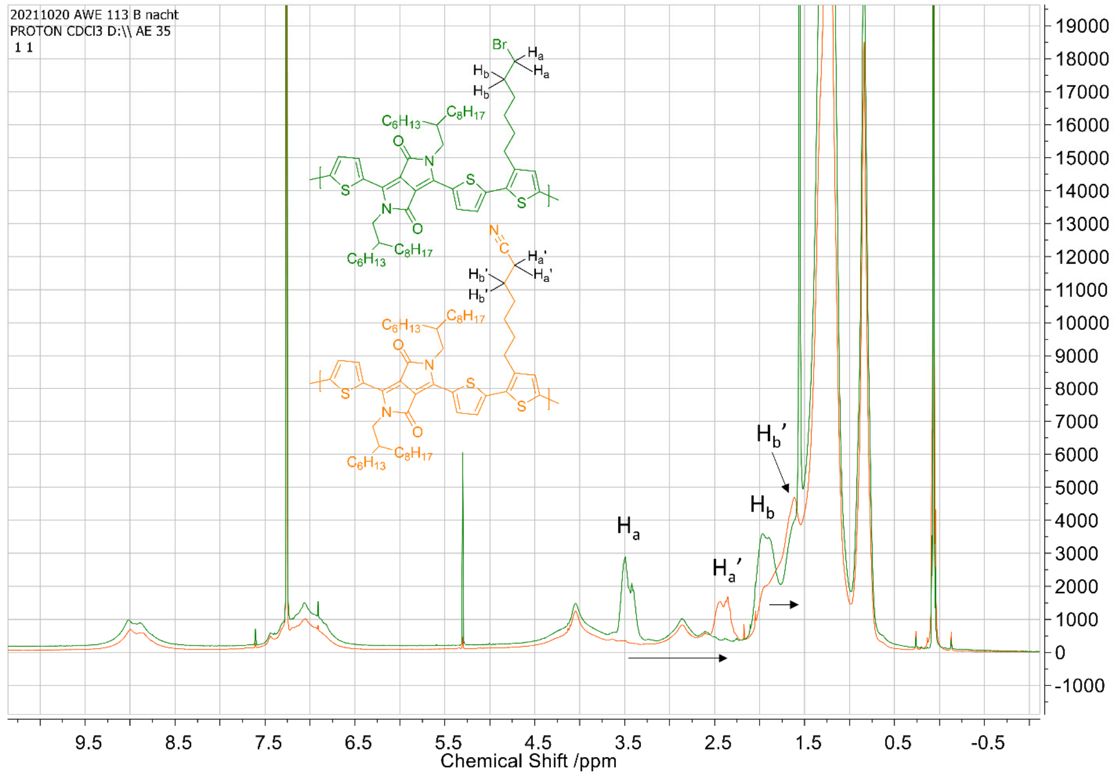

Fig. S16. 1H-NMR spectra of the brominated precursor polymer PDPP[T]$_2${2-HD}$_2$-T{BrH} and the reaction product PDPP[T]$_2${2-HD}$_2$-T{CNH}. Full conversion is evident, based on the quantitative shift of the signals H_a and H_b to H_a’ and H_b’.

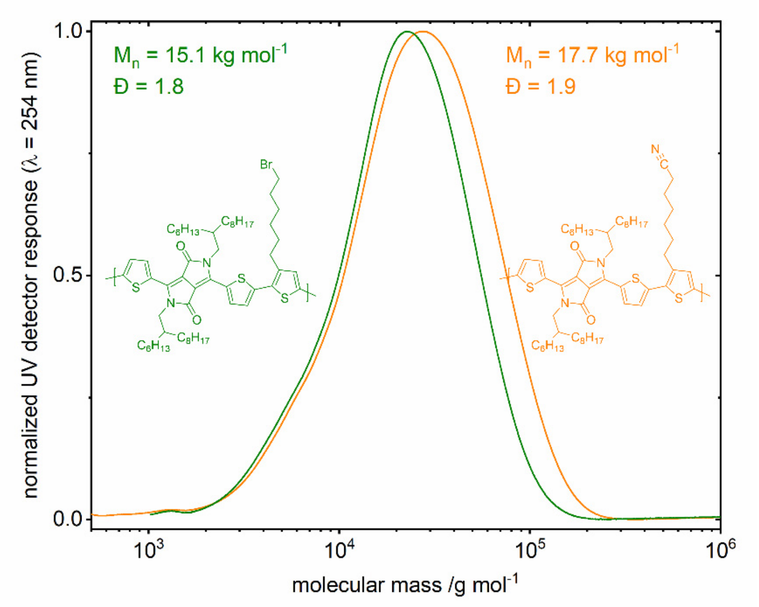

Fig. S17. GPC elugrams (Eluent: THF + 0.25 wt.% TBAB, PS calibration) and structures of PDPP[T]$_2${2-HD}$_2$-T{BrH} and PDPP[T]$_2${2-HD}$_2$-T{CNH}.

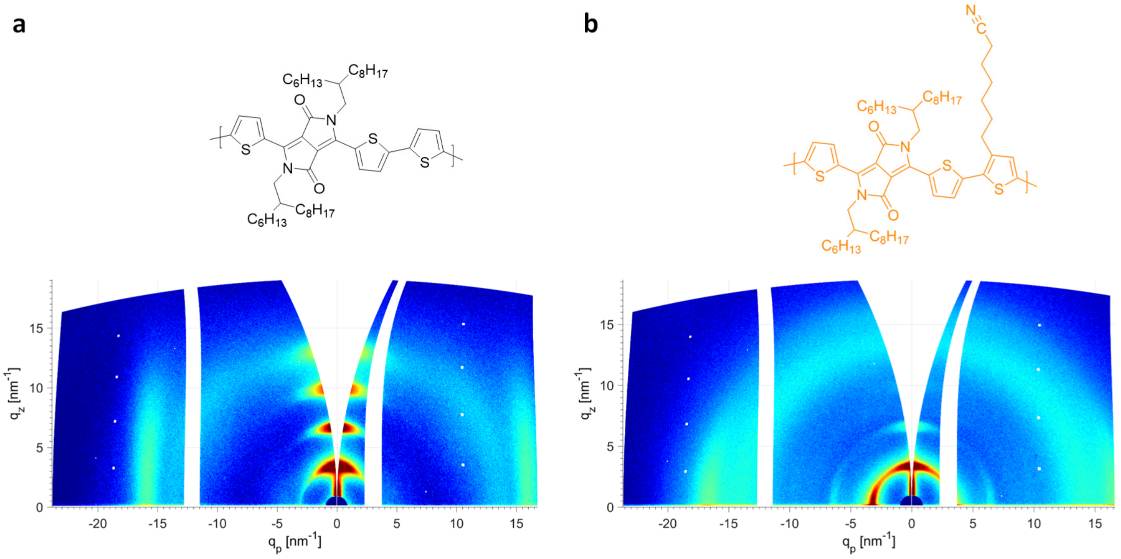

Fig. S18. The GIWAXS patterns of ~100 nm thick films of PDPP[T]$_2$-T (**a**) and PDPP[T]$_2${2-HD}$_2$-T{CNH} (**b**), both spin-coated on silicon substrates and after ordering during cooling from the melt at 10 °C·min^{-1}. While PDPP[T]$_2$-T is almost exclusively edge-on oriented, PDPP[T]$_2${2-HD}$_2$-T{CNH} clearly shows a mixed edge-on and face-on orientation. Thus, these results indicate that the addition of the hexyl side chain with the cyano group at its end to PDPP[T]$_2$-T results in the induction of face-on orientation in PDPP[T]$_2${2-HD}$_2$-T{CNH} on silicon.

SI References

1. J. B. Howard, S. Noh, A. E. Beier, B. C. Thompson, Fine Tuning Surface Energy of Poly(3-hexylthiophene) by Heteroatom Modification of the Alkyl Side Chains. *ACS Macro Lett.* 4, 725−730 (2015).
2. A. Schmitt, S. Samal, B. C. Thompson, Tuning the surface energies in a family of poly-3-alkylthiophenes bearing hydrophilic side-chains synthesized via direct arylation polymerization (DArP). *Polym. Chem.* 12, 2840–2847 (2021).
3. D. Li, A. W. Neumann, Equation of state for interfacial tensions of solid-liquid systems. *Adv. Colloid Interfac.* 39, 299-345 (1992).
4. P. Schmode, K. Schötz, O. Dolynchuk, F. Panzer, A. Köhler, T. Thurn-Albrecht, M. Thelakkat, Influence of ω-bromo substitution on structure and opto-electronic properties of homopolymers and gradient copolymers of 3-hexylthiophene. *Macromolecules* 53, 2474–2484 (2020).
5. C. J. Mueller, C. R. Singh, M. Fried, S. Huettner, M. Thelakkat, High bulk electron mobility diketopyrrolopyrrole copolymers with perfluorothiophene. *Adv. Funct. Mater.* 25, 2725–2736 (2015).
6. NIST Computational Chemistry Comparison and Benchmark Database, NIST Standard Reference Database Number 101, Release 22, May 2022, Editor: Russell D. Johnson III.
7. O. Dolynchuk, P. Schmode, M. Fischer, M. Thelakkat, T. Thurn-Albrecht, Elucidating the Effect of Interfacial Interactions on Crystal Orientations in Thin Films of Polythiophenes. *Macromolecules* 54, 5429–5439 (2021).
